# Integrative multi-omics QTL colocalization maps regulatory architecture in aging human brain

**DOI:** 10.1101/2025.04.17.25326042

**Published:** 2025-05-06

**Authors:** Xuewei Cao, Haochen Sun, Ru Feng, Rahul Mazumder, Carlos F Buen Abad Najar, Yang I. Li, Philip L. de Jager, David Bennett, The Alzheimer’s Disease Functional Genomics Consortium, Kushal K. Dey, Gao Wang

**Affiliations:** 1 Center for Statistical Genetics, The Gertrude H. Sergievsky Center, Columbia University, New York, NY, USA; 2 Computational and Systems Biology, Sloan Kettering Institute, Memorial Sloan Kettering Cancer Center, New York, NY, USA; 3 Operations Research Center, Massachusetts Institute of Technology, Cambridge, MA, USA; 4 Sloan School of Management, Massachusetts Institute of Technology, Cambridge, MA, USA; 5 Section of Genetic Medicine, University of Chicago, Chicago, IL, USA; 6 Department of Human Genetics, University of Chicago, Chicago, IL, USA; 7 Chan Zuckerberg Chicago, Chicago, IL, USA; 8 Department of Neurology, Columbia University, New York, NY, USA; 9 Center for Translational & Computational Neuroimmunology, Columbia University, New York, NY, USA; 10 Taub Institute for Research on Alzheimer’s Disease and the Aging Brain, Columbia University, New York, NY, USA; 11 Rush Alzheimer’s Disease Center and Department of Neurological Sciences, Rush University Medical Center, Chicago, IL; 12 Physiology, Biophysics and Systems Biology, Weill Cornell Medicine, New York, NY, USA; 13 Gerstner Sloan Kettering Graduate School of Biomedical Sciences, New York, NY, USA

## Abstract

Multi-trait QTL (xQTL) colocalization has shown great promises in identifying causal variants with shared genetic etiology across multiple molecular modalities, contexts, and complex diseases. However, the lack of scalable and efficient methods to integrate large-scale multi-omics data limits deeper insights into xQTL regulation. Here, we propose *ColocBoost*, a multi-task learning colocalization method that can scale to hundreds of traits, while accounting for multiple causal variants within a genomic region of interest. *ColocBoost* employs a specialized gradient boosting framework that can adaptively couple colocalized traits while performing causal variant selection, thereby enhancing the detection of weaker shared signals compared to existing pairwise and multi-trait colocalization methods. We applied *ColocBoost* genome-wide to 17 gene-level single-nucleus and bulk xQTL data from the aging brain cortex of ROSMAP individuals (average N=595), encompassing 6 cell types, 3 brain regions and 3 molecular modalities (expression, splicing, and protein abundance). Across molecular xQTLs, *ColocBoost* identified 16,503 distinct colocalization events, exhibiting 10.7(±0.74)-fold enrichment for heritability across 57 complex diseases/traits and showing strong concordance with element-gene pairs validated by CRISPR screening assays. When colocalized against Alzheimer’s disease (AD) GWAS, *ColocBoost* identified up to 2.5-fold more distinct colocalized loci, explaining twice the AD disease heritability compared to fine-mapping without xQTL integration. This improvement is largely attributable to *ColocBoost*’s enhanced sensitivity in detecting gene-distal colocalizations, as supported by strong concordance with known enhancer-gene links, highlighting its ability to identify biologically plausible AD susceptibility loci with underlying regulatory mechanisms. Notably, several genes including *BLNK* and *CTSH* showed sub-threshold associations in GWAS, but were identified through multi-omics colocalizations which provide new functional support for their involvement in AD pathogenesis.

## Introduction

Quantitative trait loci mapping of molecular phenotypes (xQTL) has been highly informative in dissecting the regulatory mechanisms underlying complex disease susceptibility loci^1–9^. Statistical colocalization methods have been instrumental in identifying causal variants shared across molecular phenotypes and complex diseases, further facilitating the prioritization of candidate causal genes and relevant cell types or tissues. However, as xQTL studies expand to encompass diverse molecular modalities, cell types, and tissues, existing colocalization methods have struggled to adequately address the resulting analytical challenges^10–12^. Existing colocalization methods are limited in their ability to handle multiple causal variants and scale to large numbers of traits. For example, the widely used COLOC^13,14^ method allows for multiple causal variants but is restricted to pairwise trait analysis, while HyPrColoc^15^, despite scaling to many traits, is limited to single causal variant per trait per gene. Other methods like eCAVIAR^16^, MOLOC^17^, Sherlock^18^, Primo^19^ are constrained in both computational scalability and the complexity of causal configurations they can accommodate. Critically, in our empirical analyses, these approaches have consistently struggled to capture gene-distal GWAS-xQTL colocalizations. These limitations underscore the need for a more versatile framework that scales to many traits, accommodates multiple causal variants, and robustly detects subtle gene-distal effects in order to fully leverage growing resources of heterogeneous, high-dimensional multi-omics data.

Here we propose *ColocBoost*, a new multi-task learning colocalization method that efficiently scales to tens or hundreds of traits while accommodating multiple causal variants within genomic regions of interest. *ColocBoost* employs an iterative gradient boosting framework in which weak learners are applied in a coupled manner across traits at each boosting round. Putative causal variants are identified through linkage disequilibrium (LD) proximity smoothing to account for correlation among nearby variants. We extensively benchmark *ColocBoost* against existing colocalization methods (COLOC^13,14^, MOLOC^17^, HyPrColoc^15^, OPERA^20^) using real-world data informed simulations, ensuring robust performance across a wide range of practical and theoretical conditions. We applied *ColocBoost* to the newly developed FunGen-xQTL resource from the Alzheimer's Disease Sequencing Project (ADSP) Functional Genomics Consortium (FunGen-AD)^21,22^, analyzing 17 gene-level molecular traits measured in range from 226 to 784 (average N=595) post-mortem donors^23,24^, encompassing 3 brain tissues, 6 brain cell types, and 3 gene-level molecular modalities (expression, splicing, and protein abundance). *ColocBoost* was deployed in both disease-agnostic (xQTL-only) and disease-prioritized settings for Alzheimer’s disease (AD) GWAS (GWAS-xQTL). We demonstrate improved performance of *ColocBoost* over existing methods on (i) disease heritability enrichment of identified variants (xQTL-only) across 57 relatively independent (genetic correlation $r_{g}<0.7$) diseases and traits (average N=305K)^25^ using Stratified LD Score Regression^26,27^ (S-LDSC), and (ii) enrichment of regulatory element-gene links validated by CRISPRi screening assays^28–31^ and promoter-capture Hi-C^32,33^. In the AD GWAS-prioritized analysis, *ColocBoost* provides functional insights into numerous GWAS loci by leveraging rich multi-omics data across molecular layers. In addition to refining loci with genome-wide significance, it further improves the identification of putative causal variants at sub-threshold GWAS loci, revealing regulatory mechanisms with support by orthogonal evidence from the ENCODE-rE2G enhancer-gene catalogs^34^.

## Results

### Overview of *ColocBoost*

**Figure 1.**
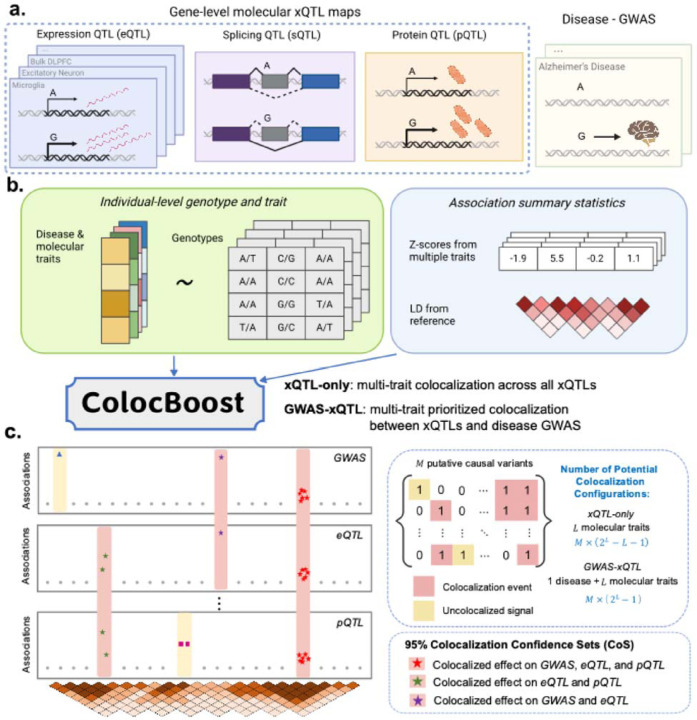
Overview of *ColocBoost* for multi-trait colocalization. **a.** *ColocBoost* uses a multi-task gradient boosting model with proximity smoothing to identify colocalized variants across multiple molecular traits (demonstrated here with gene expression, splicing and protein abundance traits) and when applicable, further integrating GWAS (demonstrated here with Alzheimer’s GWAS). **b.** *ColocBoost* takes as input either (i) individual-level genotype and trait data, or (ii) summary-level association statistics for different traits with a well-matched LD reference panel, and performs analysis in either of the xQTL-only and GWAS-xQTL modes. **c.** By allowing for multiple causal variants $\left(M\right)$ and molecular traits $\left(L\right)$, xQTL-only and GWAS-xQTL *ColocBoost* can accommodate up to $M\times \left(2^{L}-L-1\right)$ and $M\times \left(2^{L}-1\right)$ potential variant-trait causal configurations. In the schematic, distinct colocalization signals (red) are represented by 95% colocalization confidence sets (CoS) associated with traits sharing this signal, each capturing a putative causal variant with 95% probability. Uncolocalized signals unique to specific traits (yellow) are distinguished by *ColocBoost* from the colocalized events. More details on *ColocBoost* model and algorithm can be found in Methods and Supplementary Figure S1.

*ColocBoost* is a multi-trait colocalization method that (i) accommodates multiple variants with shared causal genetic effects at a locus, (ii) scales efficiently to tens or hundreds of traits, and (iii) accurately detects colocalization events for variants with relatively weak effects (Table 1). *ColocBoost* formulates colocalization as a multi-task regression problem for L traits, each with phenotype (centered at zero) and standardized genotype data ${\left\{\left(Y_{l},X_{l}\right)\right\}}_{l=1}^{L}$, and an effect-size vector $\beta_{l}=\left(\beta_{l,1},\cdots ,\beta_{l,P}\right)$ for P variants. We assume $Y_{l}∼N\left(X_{l}\beta_{l},\sigma_{l}^{2}{Ι}_{N_{l}}\right)$ where $N_{l}$ is the sample size for trait l. A variant j is considered *colocalized* if it has non-zero effect size $\left(\beta_{jl}\neq 0\right)$ for two or more traits (Methods and Supplementary Note). We introduce two complementary implementations of *ColocBoost*: a *disease-agnostic* mode (implemented here for xQTL-only integration) and a *disease-prioritized* mode (implemented here for GWAS-xQTL colocalization), where the latter focuses on a focal trait of interest—typically from a disease GWAS study (Figure 1). For L traits and M putative causal variants, *ColocBoost* effectively explores $M\times \left(2^{L}-1\right)$ variant-trait *configurations* in the xQTL-only setting and $M\times \left(2^{L}-L-1\right)$ in the GWAS-xQTL setting, representing a search space which is an order of magnitude larger than existing methods. Despite this expanded scope, *ColocBoost* remains computationally efficient, typically completing inference within minutes even for tens to hundreds of traits, making it well suited for large-scale integrative QTL applications (Table 1).

To avoid exhaustive enumeration of all possible variant-trait configurations, *ColocBoost* employs an iterative gradient-boosting algorithm^35^ built with specialized coupled weak learners, termed *Single-Effect Couplers* (SEC) which incrementally refines effect estimates to capture subtler or partial signals at each boosting round (Figure S1). Each SEC models one putative causal variant by (i) attempting to “couple” updates across traits when there is sufficient statistical evidence of shared causal effects, and (ii) for each trait, applying local LD smoothing to account for uncertainty in identifying the causal variant. Specifically, at iteration k, a SEC selects the *best update variant* $j_{k}^{*}$ and a corresponding set of traits $T\left(j_{k}^{*}\right)$ with evidence of coupled effect at the variant. The model then updates the effect size estimates as follows,

$$\beta_{l}^{k+1}=\beta_{l}^{k}-\eta_{k}SEC_{k}\left(j_{k}^{*}\left|X_{l,j},r_{l}^{k}\right.\right);\;\;\;\;\;SEC_{k}\left(j_{k}^{*}\left|X_{l,j},r_{l}^{k}\right.\right)=\omega_{l,j}\left(j_{k}^{*};r_{l}^{k}\right)\mathrm{sgn}\left(X_{l,j}^{T}r_{l}^{k}\right),$$

where $r_{l}^{k}=\left(Y_{l}-X_{l}\beta_{l}^{k}\right)$ is residual of trait l at iteration k, $\mathrm{sgn}\left(\cdot \right)$ is the sign operator, and $\eta_{k}$ denotes the dynamic learning rate (Methods). $\omega_{l}\left(j_{k}^{*}\right)$ acts as a probabilistic simplex to distribute the estimated effects of $j_{k}^{*}$ across variants in its high LD proximity, mitigating the risk of selecting non-causal variants that merely tag the true causal variant through LD (Figure S1). The analytical form of $\omega_{l}\left(j_{k}^{*}\right)$ is determined by optimizing an entropic relaxation of the objective function at each boosting round^35^ (Methods). Summing incremental contributions of all SEC for a trait l yields the estimated causal effect size for variant j on trait l, $\beta_{l,j}=\Sigma_{k}SEC_{k}$.

A key feature of *ColocBoost* is its ability to accurately identify colocalizing traits with shared effects at a causal variant v, and perform coupled SEC updates for the selected traits, $T\left(v\right):=\left\{l:\beta_{l}\left(v\right)\neq 0\right\}$. Simply selecting traits based on a substantial change in profile log-likelihood at v can be misleading, as this may incorrectly nominate traits where v in fact tags a nearby trait-specific effect—a phenomenon known as LD-induced horizontal pleiotropy^36,37^. *ColocBoost* addresses this challenge through *delayed SEC* (D-SEC) updates, designed to remove or substantially weaken other uncoupled signals by postponing coupled updates at v to later boosting rounds while prioritizing trait-specific updates in the initial rounds (Methods, Figure S1). When integrating with GWAS data, to mitigate the risk of biasing early updates toward stronger xQTL signals in LD proximity—and should in fact colocalize with—weaker signals from the disease trait of interest, *ColocBoost* incorporates *expedited SEC* (E-SEC) as a soft prioritization strategy to colocalize variants with putative causal effects on the focal trait (Methods, Figure S1). This gives rise to the two complementary implementations: a disease-prioritized (GWAS-xQTL) mode that leverages E-SEC for disease-aware inference and a disease-agnostic (xQTL-only) mode that does not; however, both use D-SEC as a core strategy to mitigate confounding from LD-induced pleiotropy (Methods, Figure S1). Therefore, *ColocBoost* dynamically manages the timing of coupled updates, thereby enabling the probabilistic support of colocalization to incrementally accumulate over boosting rounds; and as a result, reduces overfitting and false positives.

At convergence of *ColocBoost* boosting algorithm, when profile log-likelihood of all traits stabilize, a modularity-based hierarchical clustering^38^ is performed to aggregate SEC that converge on the same high-LD proxies, thereby delineating different colocalization events $\left\{s\right\}$ to represent independent underlying genetic effects (Methods). *ColocBoost* characterize $\left\{s\right\}$ using three attributes $\left\{CoS_{s}\left(\alpha \right),T\left(s\right),g\left(s\right)\right\}$. First, each causal effect is summarized by a *single-effect colocalization confidence set* (*CoS*) of coverage α (default 0.95), designed to capture one true effect variant alongside its high-LD proxies,

$$CoS_{s}\left(\alpha \right)=\left\{j_{1},\cdots ,j_{p_{0}}:p_{0}=\min\left(p:\sum_{j=1}^{p}W_{\left(j\right)}^{s}\geq \alpha ;W_{\left(1\right)}^{s}\geq \cdots \geq W_{\left(P\right)}^{s}\right)\right\},$$

where $W_{j}^{s}$ is a probabilistic weight derived by aggregating relevant SEC (Methods, Figure 1, Figure S1). Each CoS is associated with a *configuration* $T\left(s\right)$ representing the set of coupled traits sharing the causal effect $CoS_{s}$ at the locus of gene $g\left(s\right)$. We perform an additional postprocessing to remove colocalization events with low “*purity*”^36^—defined as minimum absolute correlation $\left({\left|r\right|}_{min}\right)$ between all pairs of variants within CoS—or exhibit low marginal contribution to the overall profile loglikelihood of traits in $T\left(s\right)$ conditional on other CoS (Methods). Secondly, we define *Variant Colocalization Probability* (VCP) by summing the weights over all distinct s for variant j, $VCP_{j}=1-\prod_{s=1}^{S}\left(1-W_{j}^{s}\right)$. CoS and VCP serve as analogs of the credible set (CS) and posterior inclusion probability (PIP) in single-trait statistical fine-mapping^3,39,40^. In xQTL studies where *ColocBoost* is applied separately for each gene, a single variant may receive multiple VCP values corresponding to multiple *cis*-genes. We therefore introduce MaxVCP as a variant-level annotation score, which assigns each variant its highest VCP across all genes—this is analogous to the MaxCPP annotation in fine-mapping (Methods)^41^.

**Figure 2.**
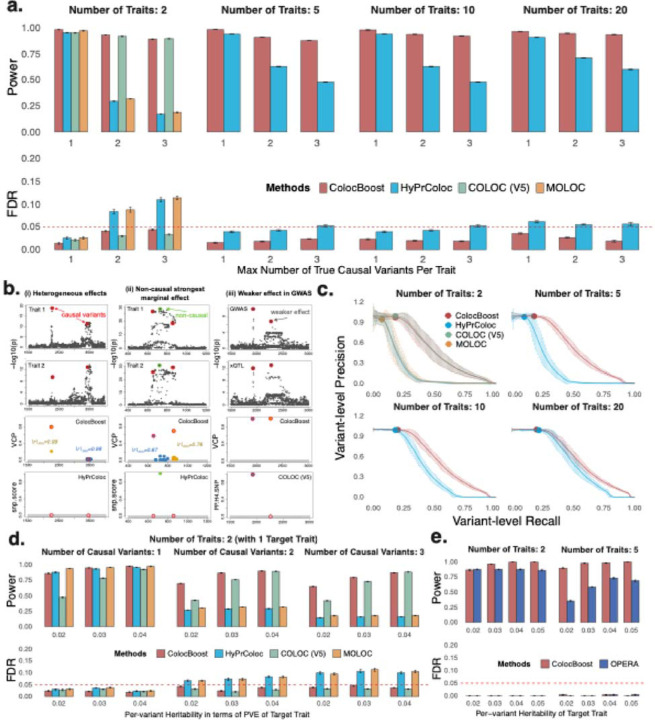
Performance comparison of *ColocBoost* with other multi-trait colocalization methods in simulation benchmarks. **a.** Statistical power and False Discovery Rate (FDR) comparisons of *ColocBoost* against COLOC (V5), MOLOC and HyPrColoc, in simulation settings involving 2, 5, 10, and 20 traits, up to five causal variants per trait per locus, with genotype data and induced colocalization configurations designed to mimic real-world xQTL datasets. Details of power and FDR calculations are provided in Methods. The X-axis represents the maximum number of causal variants across traits. **b.** Representative simulation examples illustrating limitations of competing methods: HyPrColoc with “one-causal-variant-per-trait” assumption fails to detect causal signals under (i) heterogeneous effects distribution across traits and (ii) where another non-causal variant with shared LD with causal variants has strongest marginal effect; (iii) COLOC (V5) shows reduced sensitivity to weak causal effects in the “disease-like” trait. **c.** Variant-level precision-recall curves by varying the colocalization score threshold (*ColocBoost* uses VCP; COLOC/HyPrColoc/MOLOC use variant-level scores from their respective methods; Methods and Supplementary Note S.6). **d.** Statistical power and FDR comparisons for disease-prioritized mode of *ColocBoost* (GWAS-xQTL) where there is a simulated “disease trait”, for which we lower the per-SNP heritability in the locus relative to other molecular traits to reflect expectations from real world applications. **e.** Statistical power and FDR comparison of *ColocBoost* with OPERA for GWAS colocalization, evaluated at the gene level (Methods). The red dashed line in panels **a**, **d**, and **e** denote the FDR threshold 0.05 corresponding to 95% CoS. Numerical results are reported in Supplementary Data.

*ColocBoost* offers several advantages over existing colocalization methods. While COLOC (V5)^14^ leverages the SuSiE^39^ fine-mapping framework to accommodate multiple causal variants, it is limited to two traits. Moreover, its reliance on separate *a priori* fine-mapping for each trait reduces sensitivity to weaker colocalization signals—such as gene-distal QTLs—where the marginal GWAS association may not reach stringent multiple-testing significance (Figure 2b). In contrast, *ColocBoost* overcomes this limitation by boosting SEC across traits, enabling weak-effect variants in one trait to leverage stronger effects in another, thereby enhancing detection power. HyPrColoc^15^ is fast and scales to many traits, but its assumption of at most one causal variant per trait not only reduces power, but result in high false discovery rate (FDR) when multiple causal signals are present (Figure 2b), a scenario increasingly supported by functional evidence^42^. OPERA^20^ can scale to around ten traits and relies on pairwise colocalizations “stitched” at the gene level in a meta-analysis framework, without pinpointing how many or which causal variants underlie each gene. This hinders mechanistic insights, as pinpointing causal variants is crucial for biological interpretation and follow up functional studies. Additionally, OPERA requires a target trait, typically a GWAS, therefore not compatible for xQTL-only analysis where colocalization may occur across an arbitrary subset of molecular traits. Overall, *ColocBoost* provides a scalable and flexible framework with no need to pre-specify the maximum number of causal variants (a common requirement in other multi-causal variant methods), and remains effective at identifying colocalized variants with relatively weak association signals. Further computational details of *ColocBoost* are provided in Methods and Supplementary Note. An open-source R package implementing *ColocBoost*, along with documentation and example vignettes, is available under Code Availability.

### *ColocBoost* outperforms existing colocalization methods in simulation studies

We compared *ColocBoost* against three established colocalization methods, COLOC (V5)^13,14^, HyPrColoc^15^, and MOLOC^17^, in a realistic simulation design encompassing multiple scenarios varying in the number of causal variants, variant-trait causal configurations, and effect-size heterogeneity (Figure S2a–i). We considered whole-genome sequencing data of 1,162 donors from The Religious Orders Study and Memory and Aging Project (ROSMAP), focusing on *cis* loci (TSS ± 1.5Mb) of 1,287 unique genes, where each gene was sampled from a distinct topologically associating domain (TAD) out of 1,381 human brain TADs genome-wide^43^. We simulated 2, 5, 10, 20, and 50 molecular traits per gene, ascertaining the effect size of each variant such that it explains a fixed proportion of the trait variance at 5%, and allowing up to five causal variants per trait per locus (Methods). For each causal variant, we simulated its colocalization trait configuration by randomly selecting a subset of traits, with the number of colocalized traits determined by the distribution of overlapping genome-wide fine-mapping results from 62 FunGen-xQTL contexts^22^. Statistical power was defined as the proportion of correctly identified 95% CoS that contain the true causal variant, weighted by the number of true colocalizing traits. False discovery rate (FDR) was computed as the fraction of detected CoS that either failed to capture a true causal variant or incorrectly incorporated at least one non-colocalizing trait (Methods). Our definitions of power and FDR are intentionally stringent, penalizing both the incorrect assignment of colocalized sets and any misattribution of traits within those sets. Since CoS is not a native metric to competing methods, we extended each method to define the best analogous CoS (Methods). Additionally, we conducted variant-level benchmarking by treating variant-level posterior probabilities (from COLOC, HyPrColoc, and MOLOC) or VCP (from *ColocBoost*) as comparable measures of colocalization evidence (Methods).

Across different simulation scenarios, *ColocBoost* consistently achieved high statistical power (averaging 0.934) while controlling the FDR below the expected 5% for CoS-level benchmarks (Figure 2a). In pairwise colocalization settings with multiple causal variants, *ColocBoost* exhibited 2.9 to 5.2-fold higher power over HyPrColoc and MOLOC respectively, while maintaining significantly lower FDR. *ColocBoost* and COLOC (V5) showed comparable performance under this setting. For 5, 10, and 20 traits, we compared *ColocBoost* only with HyPrColoc due to scalability constraints of COLOC (V5) and MOLOC (Table 1). Even in a one-causal-variant regime which aligns with the HyPrColoc model assumptions, *ColocBoost* attained higher power and lower FDR (Figure 2a). Under multiple causal variants, *ColocBoost* expectedly further outperformed HyPrColoc, demonstrating up to 73% higher power and 60% lower FDR, with performance gains increasing proportionally to the number of causal variants (Figure 2a, Figure S2b). We underscore two representative multiple causal variant simulation scenarios where HyPrColoc underperforms in unexpected ways: (i) heterogeneous effects across traits where HyPrColoc fails to detect either causal variant despite typically being expected to recover at least one under a single-causal assumption; and (ii) a non-causal variant in shared LD with two causal variants and having the strongest marginal association, HyPrColoc admits false positives driven by marginal signal strength (Figure 2b–i, 2b–ii). *ColocBoost* can resolve the locus and identify the two causal variants in both cases. We also compared *sizes* and *purity* of 95% CoS among methods as benchmark for how precisely true causal variants are colocalized (Methods), and found that all methods produced CoS with average variant counts under 10 and maintained purity above 0.95 (Figure S2c). When the number of traits increased to 50, *ColocBoost* continued to show improved power and significantly better controlled FDR compared to HyPrColoc in both single and multiple causal variant scenarios (Figure S2d). Additionally, we benchmarked *ColocBoost* against HyPrColoc across four multi-trait simulation designs adopted in HyPrColoc manuscript^15^ (Figure S2a–ii, Supplementary Note S.6), along with a fully colocalized scenario where multiple causal variants affect all traits. In all cases, *ColocBoost* outperformed HyPrColoc in power while HyPrColoc showed inflated FDR under multiple causal variants setting (Figure S2e,f), particularly in the fully colocalized scenario. As a sanity check, we also evaluated a null scenario with no causal variants across any trait; both methods maintained well-controlled Type I error rates (T1E < 0.005; Figure S2g).

Next, under the primary simulation design informed by FunGen-xQTL data, we evaluated variant-level performance of the above methods by comparing the precision-recall curves generated by varying the threshold of variant-level colocalization probability from 0 to 1 and computing area under the precision-recall curve (AUPRC) (see Methods for variant-level probability scores of each method). *ColocBoost* outperformed HyPrColoc and MOLOC, achieving 2.56±0.22 and 2.48 0.22-fold higher AUPRC respectively across all simulation scenarios (Figure 2c). In the two-trait scenario, *ColocBoost* showed similar precision-recall as COLOC (p=0.41, two-sided *t*-test). While variant-level comparisons are informative for benchmarking, we caution against focusing on a single top-ranked variant for any colocalization method: in regions of strong LD, such a variant can be merely a proxy of the true causal variant, thus constituting false discoveries at variant level (Figure S3a). Consequently, we recommend evaluations based on 95% CoS as they offer more robust inference by accounting for uncertainties due to LD.

In practice, it is often of interest to colocalize a disease GWAS with multiple molecular QTL traits to elucidate the functional basis of disease associations. An important technical aspect of this colocalization is that most GWAS traits tend to have significantly lower per-variant contribution to heritability compared to xQTL on molecular traits. To simulate this scenario, we create a “disease-like trait” with per-variant heritability varying between 2% and 4% (while such per-variant heritability is relatively large for a disease GWAS, we note that real-world GWAS typically benefits from much larger sample sizes to detect weaker effects with comparable power). We then applied *ColocBoost* in the GWAS-xQTL mode using E-SEC on the disease trait. In the two-trait scenario with high per-variant heritability, *ColocBoost* showed similar performance with COLOC (V5). However, when the disease trait had lower per-variant heritability, *ColocBoost* demonstrated greater advantage in both power and FDR, demonstrating its capacity to detect subtler signals (Figure 2d; with an illustrative example in Figure 2b–iii). We next compared *ColocBoost* to the recently proposed OPERA^20^ method designed for GWAS-centric multi-trait colocalization. Since OPERA only offers gene-level outputs, we defined a parallel gene-level metric for *ColocBoost* to enable a fair comparison (Methods). While both methods controlled FDR well below 0.05 in this setting, GWAS-xQTL *ColocBoost* achieved up to 2.53-fold higher power compared to OPERA, with performance gains increasing proportionally to the number of traits and the number of causal variants involved (Figure 2e, Methods). Moreover, *ColocBoost* demonstrated improved power (at well controlled FDR) over OPERA under an additional simulation setting where OPERA has been reported to show excellent performance^20^ (Figure S2a–ii, Figure S3b).

Overlapping samples are common in xQTL analyses spanning multiple modalities from donors of the same cohort. Since this can induce dependencies (correlated residuals independent of genetic effects) across traits, in principle, accounting for such correlations may improve method performance. However, Foley *et al.*^15^ showed that treating studies as independent typically yielded controlled FDR and robust power in capturing colocalizing traits while conferring an advantage of reduced computational complexity. To validate this, we considered extensions of the multi-trait simulation design in Figure 2a, incorporating realistic cross-trait correlations estimated from FunGen-xQTL data (Figure S2a–iii, Supplementary Note S.6). Despite not modeling these correlations by default, *ColocBoost* maintained comparable power and FDR across all simulation scenarios (Figure S3c,d). While we recommend the current *ColocBoost* implementation for its balance of performance and scalability to many traits, we provide technical details to incorporate sample overlap adjustments within *ColocBoost* (Supplementary Note S.2.5).

Finally, we performed three secondary analyses to assess *ColocBoost* under specialized or less typical circumstances. First, we considered a scenario where each trait has available association summary statistics but not the matching LD reference. In this scenario, like fine-mapping, a single-causal-variant assumption without LD-proximity smoothing was used for colocalization, under which *ColocBoost* continued to exhibit substantially better FDR control with slight loss of power compared to HyPrColoc (Supplementary Note S2.5; Figure S3e). Second, we explored a version of *ColocBoost* with only one trait, referred to as *FineBoost*, to compare its fine-mapping performance with SuSiE^39^. Even though *FineBoost* is primarily a special case of *ColocBoost* rather than a dedicated fine-mapping tool, it matched SuSiE in power while more effectively controlling FDR in scenarios featuring multiple (up to five) causal variants, thereby underscoring its robustness as a statistically rigorous model for both colocalization and single-trait genetic association studies (Figure S3f). Third, we tested different choices of the probabilistic simplex $\omega_{l}$ by varying the way in which it integrates marginal association evidence with proximal LD. We observed an overall robust performance of *ColocBoost* to these choices, with slightly better performance under the default choice (Methods; Figure S4).

We conclude that *ColocBoost* demonstrates robust performance over existing colocalization methods across diverse simulation designs, including multiple causal variants, large sets of traits, complicated colocalization patterns, lower causal effect sizes, overlapping samples, and absence of LD data.

### xQTL-only *ColocBoost* decodes molecular regulatory architecture in aging brain

**Figure 3.**
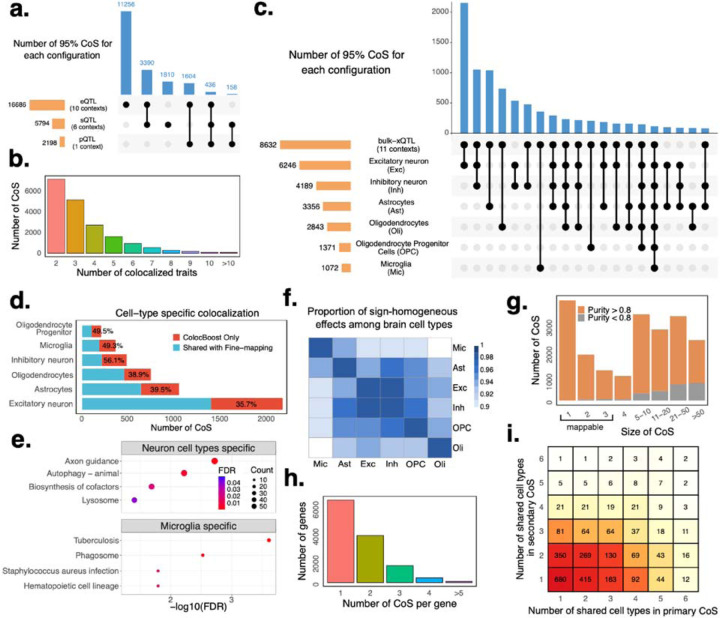
*ColocBoost* xQTL analysis across cell types and traits modalities. *ColocBoost* was applied to 17 gene-level cis-xQTL datasets from the aging brain cortex of ROSMAP subjects (average N=595) spanning 16,928 genes. **a**. UpSet plot summarizing the colocalization patterns across three different molecular trait modalities (expression, splicing, protein abundance). See Table 2 for details of each modality. **b**. Distribution of the number of 95% CoS corresponding to the different numbers of colocalized traits per locus; we omit from consideration 0.55% loci with more than 10 colocalized traits. **c**. UpSet plot summarizing the colocalization patterns across 6 pseudo-bulk brain cell-type eQTL data, along with bulk-xQTL data. We highlight putative causal variants with (i) shared effect across multiple brain cell types, and (ii) cell-type specific effect obtained through colocalization between bulk xQTL and pseudo-bulk eQTL for that cell type. **d**. The fraction of cell-type specific colocalizations (measured at the level of CoS) for different brain cell types recovered by fine-mapping credible set within the cell type. **e**. Top 4 significantly enriched (FDR<0.05) pathways (using enrichKEGG^59^) for eGenes linked to cell-type specific colocalizations in excitatory neurons and microglia. Size of the circle denotes the number of eGenes matched to the pathway and the color denotes the level of FDR-adjusted significance. **f**. For each pair of brain cell types, we assess the homogeneity in estimated causal effects directions by measuring the proportion of colocalized signals exhibiting concordant effect size signs. **g**. Empirical distribution of the number of 95% CoS in terms of the number of variants each CoS contained. CoS are color-coded by *purity*, with high-purity sets (*purity* > 0.8) distinguished from moderate-purity sets (0.5 < *purity* ≤ 0.8). **h**. Distribution of the number of CoS per gene; only 0.8% genes harbor more than four colocalized sets. **i**. For each gene with multiple CoS, we consider a pair of primary CoS and secondary CoS, and evaluate the number of brain cell type pseudo-bulk eQTLs showing shared effects for each CoS in the pair. We present a heatmap of the number of pairs of primary and secondary CoS for the same gene, showing different patterns of sharing across brain cell type eQTLs. Numerical results are reported in Supplementary Data.

We conducted a genome-wide xQTL-only *ColocBoost* analysis across 17 cis-xQTL traits from the aging brain cortex of ROSMAP subjects (average N=595; Table 2)^22,24,44–46^, spanning 16,928 genes and three gene-level molecular trait modalities including gene expression, splicing and protein abundance. Our data consist of 6 pseudo-bulk expression QTL (eQTL) datasets derived from different cell types in brain dorsolateral pre-frontal cortex (DLPFC), 4 bulk eQTL datasets spanning three brain cortical regions and CD14^+^/CD16^−^ monocytes from peripheral blood mononuclear cells (PBMCs), 6 bulk splicing QTL (sQTL) datasets comprising both productive and unproductive splicing in the same three cortical regions^47^, and one protein abundance QTL (pQTL) dataset from DLPFC^22^ (Methods, Table 2). Overall, *ColocBoost* identified 18,654 colocalization events involving 11,257 genes and 16,504 distinct 95% CoS (Table S1). At least one colocalization event was detected for 66.5% genes; of these, 27.6% represented cross-modality colocalization bridging at least two of the three modalities, and 2.3% spanned all three modalities (Figure 3a). A large fraction (61.8%) of colocalization events were shared across more than two traits (Figure 3b, Figure S5a). eQTLs and sQTLs contributed to more colocalization events compared to pQTLs, partly due to protein abundance data being available for only 7,641 of the 16,928 genes (Table 2). Restricting the analysis to 3,655 genes profiled across all three modalities yielded more balanced colocalization patterns, though eQTLs and sQTLs still demonstrated more colocalizations than pQTL (Figure S5b).

Next, we focused on xQTL-only colocalization events where the 95% CoS included at least one eQTL from a specific brain cell type. Of the 18,654 total events identified, 10,237 (55%) involved at least one cell-type eQTL (Figure 3c). Within this subset, 4,973 events (48.6%) featured colocalization between a single-cell-type eQTL and one or more bulk xQTL. Overall, 27.9% of these cell-type-specific events mapped to microglia or astrocytes, whereas 54.1% involved neuronal subtypes (Figure 3c). Notably, 35.7–56.1% of these cell-type specific colocalization signals were not captured by the matched cell-type eQTL fine-mapping using SuSiE (Figure 3d, Figure S5c), while on the other hand, 58.7–76.6% of fine-mapped cell-type eQTLs were identified to colocalize with other xQTL (Figure S5c). This underscores the utility of integrating bulk xQTL data with pseudo-bulk cell-type level data in boosting detection power for weaker cell-type level signals, as previously demonstrated for eQTL mapping^48^. To evaluate the impact of modality beyond eQTL, we conducted an ablation *ColocBoost* analysis that removed all sQTL and pQTL datasets, retaining only the 10 eQTL contexts (6 cell-type eQTL and 4 bulk eQTL). Although a large fraction (82.1%) of these cell-type involved colocalizations can be detected by including only bulk eQTL without sQTL or pQTL (Figure S5d), the remaining 17.9% represent regulatory signals that could only be identified through integrated multi-omics analysis. eGenes corresponding to neuron-specific colocalization showed significant enrichment in axon guidance pathways, while microglia-specific signals showed enrichment in infectious disease pathways^49,50^ (Figure 3e; Table S2); microglia are known to be equipped with receptors to respond to detect and respond to a wide array of pathogens^51^. We also observed a 1.36 to 10.32-fold excess-of-overlap (EOO) of the cell-type specific colocalization eGenes with cell-type gene programs from external brain single-cell RNA-seq data^25,52,53^ (Methods; Figure S5e). Among the 5,264 colocalization events spanning multiple cell types, excitatory and inhibitory neurons jointly appeared in 61.8% of them, with 99.5% of these neuronal-shared events maintaining consistent causal effect-size directions (Figure 3f). 132 colocalization events showed coupling across all six brain cell types. 69.5% of multiple cell type colocalizations involved a bulk QTL; ablating these bulk xQTLs resulted in a mere 3.6% reduction in multiple cell-type colocalizations (Table S1).

Among all *ColocBoost* 95% CoS, 3,712 (19.9%) consists of a single variant and 6,532 (35%) contain three or less variants, which we refer to as *mappable* CoS (Figure 3g). Variants in these low-complexity CoS exhibit strong functional enrichment: 10.6-fold in cV2F regulatory score^54^ and 3.31-fold in RegulomeDB annotations^55,56^ relative to the remaining CoS (Figure S6a). 84.5% of baseline-LD v2.2 annotations^57^ are significantly enriched in *mappable* CoS^58^ (Figure S6b where p-values of enrichment are estimated by Jackknife method through a leave-one-chromosome-out strategy). Notably, 45.9% of genes contain two or more colocalized events, highlighting the importance of a colocalization method capable of accommodating multiple causal variants (Figure 3h). For each gene, we designated the 95% CoS associated with the highest increase in profile log-likelihood as the *primary* colocalization and others as *secondary* (Methods, Figure S7a–b). We observed that secondary CoS were on average 2.24 times more distal from gene TSS and 44.5% more cell-type-specific compared to primary CoS (Figure 3i). Moreover, primary CoS showed significantly higher coding and promoter enrichment, especially among variants with MaxVCP > 0.5 (Figure S7c). As an illustrative example, we present *ARSB* featuring two distinct colocalization loci: the primary CoS comprises a single variant with shared effect across neuron and glial cells, whereas the secondary CoS comprises of eight variants and shared only among glial cells (Figure S7d). These discrete regulatory events suggest that *ARSB* expression may be modulated by multiple, partially independent mechanisms across different cell types.

As a secondary analysis, we applied *ColocBoost* to eQTL data from 13 GTEx brain tissues with moderate sample size (N=168, Table S3). Of the colocalizations identified in three cortical GTEx tissues—brain cortex, anterior cingulate cortex (BA24), and frontal cortex (BA9)—a substantial fraction (46.9%) overlapped with ROSMAP, whereas only 10.4% of ROSMAP colocalized events appeared in GTEx (Figure S8). This asymmetry likely arises from three factors: (i) larger sample size in ROSMAP, which increases power to detect weaker QTL, and (ii) the cell-type-level resolution available in ROSMAP, which can reveal additional layers of regulatory variation masked by bulk tissue data, and (iii) differences in donor age, with ROSMAP consisting of an aging population (average age: 88 years) versus the younger GTEx cohort (average age: 53.5 years), potentially capturing additional age-related genetic effects.

### Colocalized xQTL are concordant with CRISPR and enhancer-gene maps

**Figure 4.**
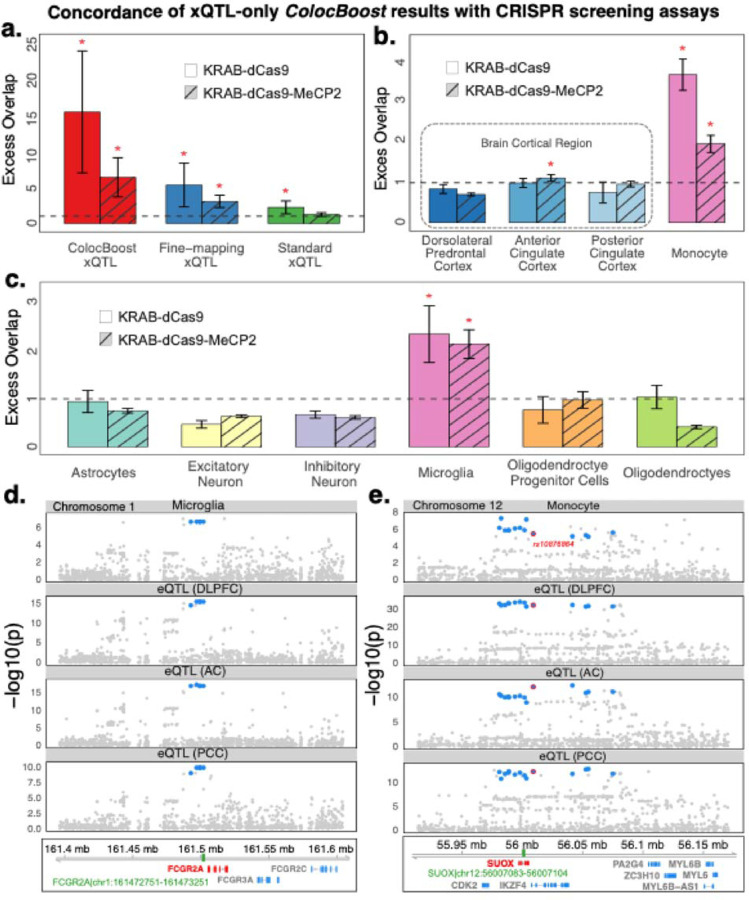
Validation of *ColocBoost* colocalization signals using CRISPR data. **a**. Variant set-level excess-of-overlap (EOO) analysis of (i) 95% CoS-gene links from xQTL-only ColocBoost, (ii) standard marginal xQTL-gene associations (FDR<0.05), merged across all 17 xQTL datasets and (iii) 95% credible set (CS)-gene links from SuSiE fine-mapping, merged across all 17 xQTL datasets (Methods), against 569 silver-standard element-gene links from 3 aggregated CRISPR interference (CRISPRi) datasets in K562 cell line using KRAB-dCas9 protocol (refs), and 8,192 silver-standard element-gene links from the recent STING-seq data in K562 using a newer KRAB-dCas9-MeCP2 protocol (ref). **b, c**. Relative EOO analysis of 95% CoS-gene links across **b.** 3 different brain cortical regions and CD14+CD16- bulk monocytes, and **c.** 6 different brain cell types, against element-gene links from aggregated CRISPR (dCas9) and STING-seq datasets. Red asterisks denote significant enrichment based on EOO test adjusted for multiple testing (Bonferroni adjusted p-value<0.05). **d**. As an illustration, a CoS-gene link from microglia-specific eQTL colocalization in *FCGR2A* is validated by CRISPRi in K562 cells. **e**. A second CoS-gene link, reflecting colocalization between monocyte eQTLs and bulk brain region data at the *SUOX* gene, is similarly validated by CRISPRi in K562 cells. The green box denotes the CRISPRi enhancer linked to the target gene. Numerical results are reported in Supplementary Data.

We systematically evaluated the concordance of CoS-gene maps implicated by *ColocBoost* based on the enrichment for regulatory element-gene links derived from orthogonal functional assays from cell lines. These benchmarking datasets include (i) 569 element-gene links from aggregated CRISPRi data (KRAB-dCas9) of 3 studies^29–31^ as used in Gschwind et al.^34^, (ii) 8,192 element-gene links from the recent STING-seq assay using an updated CRISPRi protocol (KRAB-dCas9-MeCP2)^29^ covering 500 GWAS loci across 44 blood-related traits, and (iii) 118,389 regulatory element-gene maps from Promoter Capture Hi-C assay in the dorsolateral prefrontal cortex brain tissue^32,33^, which profiles targeted 3D contacts linking gene promoters to proximal or distal regulatory elements. Specifically, we constructed 95% CoS-gene links and compared them with (i) all SNP-Gene links derived from conventional xQTL association analysis and (ii) SuSiE fine-mapped 95% credible set (CS)-gene links, merged across all 17 ROSMAP xQTLs (Figure 4a, Methods). CoS-gene pairs from xQTL-only *ColocBoost* exhibited a high 15.42-fold $\left(p=8\times 10^{-4}\right)$ and 6.38-fold $\left(p=9.8\times 10^{-5}\right)$ variant set-level EOO with aggregated CRISPRi and STING-seq links in K562 respectively, significantly exceeding the EOO observed for standard xQTL SNP-Gene and fine-mapped CS-gene links (one-sided *t*-test of difference, p=0.0024 and 0.027 respectively) (Methods; Figure 4a). Consistent with these findings, *ColocBoost* CoS outperformed xQTL CS by 1.73-fold EOO in brain cortical PCHi-C (Figure S9a).

Overall, 53 CoS-gene links were validated by at least one of the two CRISPR datasets, of which 15 (28.3%) involved immune cell type eQTLs in microglia or monocyte among the coupled traits (Figure 4b,c, Table S4). CoS-gene links in 3 brain cortical regions did not show significant EOO relative to all CoS-gene links; however, CoS-gene links in CD14+/CD16- monocytes showed high enrichment (EOO=3.71, $p=1\times 10^{-41}$ in CRISPRi and EOO=1.97, $p=8\times 10^{-19}$ in STING-seq; Figure 4b). Concordant with this observation, out of the 6 brain cortical cell types, only CoS-gene links involving microglia were uniquely enriched (EOO=2.35, $p=8\times 10^{-6}$ in CRISPRi and EOO=2.14, $p=6\times 10^{-14}$ in STING-seq; Figure 4c). This confirms the expected regulatory concordance between K562 and immune cell types in the brain, while also highlighting its discordance with broader brain cell types. Although K562 is an erythroleukemia cell line that does not closely match most brain cell types, it remains the most extensively studied line for CRISPR screening assays containing hundreds of element-gene links, providing reasonable statistical power for benchmarking purpose in our work. We assessed concordance between CoS-gene links involving different brain cell types and bulk brain regions against the brain PCHi-C links, and found significant relative EOO in oligodendrocyte progenitor cells and oligodendrocytes, but not in any bulk brain region (Figure S9b,c).

An illustrative example of the concordance between *ColocBoost* and CRISPR data is the 95% CoS *chr1:161493252–161502998*, comprising of 5 variants mapped to *FCGR2A*^60,61^. It overlaps the regulatory element identified by CRISPRi (*chr1:161502961–161503461*) mapped to the same gene (Figure 4d). The CoS is microglia specific among single cell contexts—consistent with the role of *FCGR2A* encoding a cell-surface receptor involved in clearing immune complexes^62^—and propagates across all 3 bulk brain region eQTLs,. Another example of such concordance is a relatively broader 95% CoS mapped to *SUOX*, with 14 variants spanning *chr12:55977282–56078047* which contains the element *chr12:56007083–56007104* mapped to the same gene by CRISPRi. The CoS spans 9 molecular traits, comprising of bulk eQTL data from monocytes and 3 brain cortical regions, alternative splicing QTLs, as well as with other brain cell type eQTLs (Figure 4e, Figure S9d). One variant *rs10876864* (*chr12:56007301:G:A*) in the CoS has been previously associated with autoimmune^63^ and allergic conditions^64^.

### Colocalized xQTL variants are enriched in complex disease heritability

Genome-wide MaxVCP scores derived from our multi-omics colocalization map can serve as a new variant-level functional annotation score. To evaluating its informativeness across a broad range of complex diseases, we annotated 9,991,229 variants with minor allele counts≥5 in the 1000 Genomes Project Europeans using 5 different MaxVCP derived features: (i) *MaxVCP*-*xQTL*, based on xQTL-only *ColocBoost* analysis of all 17 ROSMAP xQTL datasets; (ii) *MaxVCP*-*eQTL*, restricting the analysis to 10 eQTL traits; (iii) *MaxVCP*-*pseudobulk*, restricting the analysis to 6 cell-type pseudo-bulk eQTLs; (iv) *MaxVCP*-*bulk*, restricting the analysis to bulk eQTLs in three brain regions; and (v) *MaxVCP*-*GTEx*, obtained by applying xQTL-only *ColocBoost* to 13 GTEx brain tissues (Methods, Table S5). These five annotations showed moderate inter-correlation (average r=0.39; Figure S10a). To assess their relevance to disease heritability, we applied Stratified LD Score Regression^27^ (S-LDSC) across 57 relatively independent $\left(r_{g}<0.7\right)$ complex diseases and traits (average GWAS sample size N=305K), including 22 blood-related and 18 brain-related traits (Table S6). We compared the heritability enrichment and the standardized effect sizes $\left(\tau^{*}\right)$ of these annotations conditional on 97 baseline-LD v2.2 annotations, comprising coding, conserved, regulatory, and LD-linked features^57,65^ (Methods).

**Figure 5.**
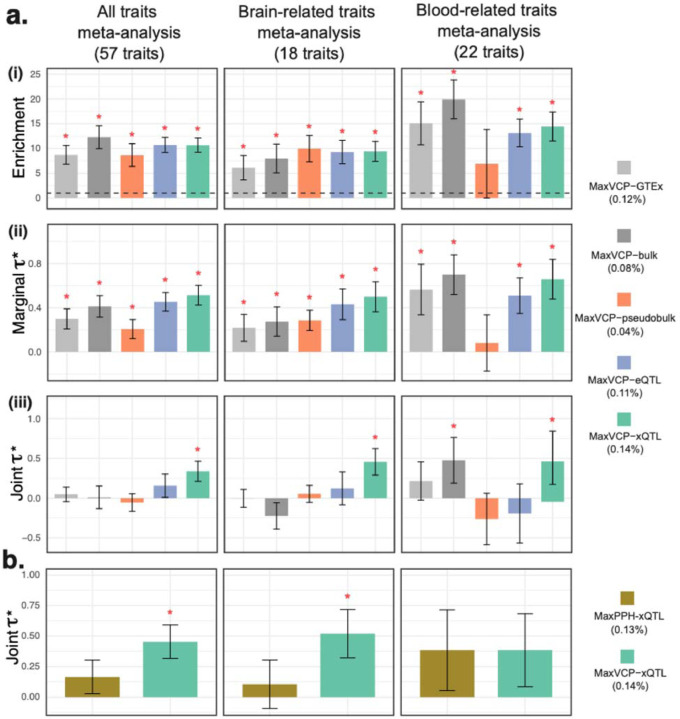
Disease heritability analyses of variant-level functional annotations derived from *ColocBoost*. **a.** We generated 5 MaxVCP variant-level functional annotation scores by performing xQTL-only *ColocBoost* (Results) and performed S-LDSC heritability analysis of the resulting annotations. **(i)** Heritability enrichment conditional on 97 baseline-LD v2.2 annotations. **(ii)** Standardized effect sizes of the 5 MaxVCP scores, each conditional on 97 baseline-LD v2.2 annotations (marginal $\tau^{*}$). **(iii)** Standardized effect sizes, jointly analyzing 97 baseline-LD v2.2 annotations and all 5 MaxVCP scores (joint $\tau^{*}$). **b.** Standardized effect sizes of the MaxVCP-xQTL and an analogous score based on the HyPrColoc method in a joint model involving other 97 baseline-LD v2.2 annotations. All results are meta-analyzed across 57 complex traits as well as subsets of 18 brain-related and 22 blood-related traits following from S-LDSC recommendations^57^. The asterisks indicate statistical significance (Bonferroni adjusted p<0.05). Error bars indicate 95% confidence intervals. Numerical results are reported in Supplementary Data.

All five MaxVCP annotations attained high heritability enrichments in meta-analysis across all 57 traits combined (average 10.6±0.74-fold), as well as across brain-related traits (Figure 5a). 4 out of 5 MaxVCP annotations—except MaxVCP-pseudobulk—showed significant heritability enrichment for blood traits meta-analysis. When conditioned on the baseline-LD annotations, MaxVCP-xQTL attained the highest $\tau^{*}$ (0.51 and 0.50 respectively) compared to other MaxVCP annotations (annotations with $\tau^{*}\geq 0.5$ are generally considered to be highly impactful for disease^41,66^). In a subsequent joint heritability enrichment analysis including five MaxVCP annotations, MaxVCP-xQTL remained significant with moderate to high values of joint $\tau^{*}$ —0.33, 0.51 and 0.46 for all traits, brain traits, and blood traits meta-analysis respectively. The consistently stronger heritability signal for MaxVCP-xQTL over other MaxVCP scores highlights the additional advantage conferred by integrating multiple QTL modalities and contexts. We next compared the heritability information of MaxVCP-xQTL against an analogous score from similarly applying HyPrColoc to all modalities of xQTL data (MaxPPH-xQTL). When considered individually, both annotations were enriched for disease heritability and exhibited high marginal $\tau^{*}$ (Bonferroni corrected p<0.05) conditioned on the baseline-LD annotations (Figure S10b). However, in the joint analysis of the two annotations, only MaxVCP-xQTL remained significant in all traits and brain traits meta-analyses (joint $\tau^{*}=0.45$ and 0.52 respectively; Figure 5b), effectively subsuming the information MaxPPH-xQTL captured. In comparison, for blood traits meta-analyses, both the annotations showed comparable joint $\tau^{*}$ signal.

We conducted five secondary analyses to evaluate robustness of MaxVCP-xQTL. First, we compared MaxVCP-xQTL with a *CoS*-*xQTL* annotation that labels each variant as 1 if it appears in a 95% CoS (Methods). CoS-xQTL showed lower heritability enrichment than MaxVCP-xQTL and was not conditionally informative when jointly analyzed with MaxVCP-xQTL (Figure S10c). Second, we tested if binarizing MaxVCP-xQTL at various thresholds affected heritability signals, finding that these discrete annotations performed comparably or worse than the continuous MaxVCP-xQTL (Figure S10d). Third, we contrasted MaxVCP-xQTL with a *MaxPIP* annotation built from maximum SuSiE fine-mapping PIP across all 17 xQTL traits. MaxVCP-xQTL showed higher standardized effect sizes both in marginal and in joint analysis, although SuSiE still contributed substantially to the joint analysis for brain-related traits. This likely reflects additional contribution from trait-specific xQTL not colocalizing with other modalities or contexts (Figure S10e). We next investigated the enrichment of MaxVCP-xQTL in variants confidently fine-mapped (PIP>0.95) from 94 UK Biobank traits^34^ and 930 Million Veteran Program (MVP) GWAS traits^67^ using an EOO approach (Methods). We observed a high 17±0.70-fold and 11.8±0.62-fold enrichment respectively (Figure S10f), and prioritized 129 such variants with MaxVCP-xQTL ≥0.5 (Table S7). Finally, we stratified MaxVCP-xQTL into cell-type-specific (colocalization of a single cell type with bulk QTL) and cell-type-shared (colocalization among multiple cell types) annotations to assess whether cell-type specificity dominates disease heritability signal. Both annotations remained jointly informative in the meta-analyses for all traits and brain traits (Figure S10g), suggesting that while cell-type specificity in colocalization is important, shared regulatory architecture also substantially contributes to disease risk.

### GWAS–xQTL *ColocBoost* identifies regulatory variants for Alzheimer's disease

**Figure 6.**
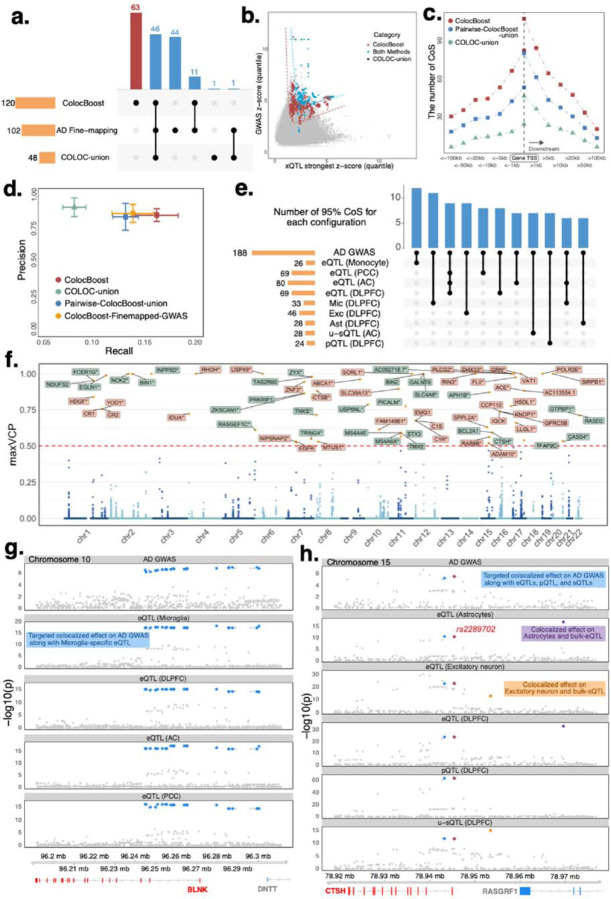
AD–xQTL *ColocBoost* identifies colocalized variants between xQTLs and AD GWAS. **a**. UpSet plot of distinct (i) 95% CoS identified by GWAS–xQTL *ColocBoost*, (ii) union of 95% CoS identified by applying COLOC on each xQTL with AD GWAS (COLOC-union), and (iii) 95% SuSiE credible sets (CS) from AD GWAS fine-mapping. **b**. Scatter plot across variants comparing the marginal association z-scores in AD GWAS against the quantile-matched z-scores corresponding to the strongest marginal association signals (in terms of the magnitude of z-scores) across all xQTL traits for each CoS. We color-code variants within CoS corresponding *ColocBoost,* COLOC-union and both methods. **c**. Distribution of distances from the gene TSS for CoS from AD–xQTL *ColocBoost*, COLOC-union, and the union of CoS identified by *ColocBoost* applied pairwise to one AD GWAS and each of xQTL traits (pairwise-ColocBoost-union). **d.** Precision-recall analysis comparing CoS-gene links from AD-xQTL *ColocBoost*, COLOC-union, pairwise-ColocBoost-union, and a version limiting AD-xQTL *ColocBoost* to AD fine-mapped variants (ColocBoost-Finemapped-GWAS) against enhancer-gene links predicted by ENCODE-rE2G across 354 biosamples. Error bars along both axis indicate 95% confidence intervals. **e**. An UpSet plot focusing showing the distinct colocalization patterns across xQTLs exhibited by 95% CoS from AD-xQTL *ColocBoost*. **f**. Manhattan plot of variant level MaxVCP from *ColocBoost*, with labeled genes containing variants with MaxVCP>0.5 and highlighting microglia contributions (green). **g.** An example case of AD-xQTL colocalization demonstrating a single CoS with cell-type specific colocalization in microglia for *BLNK* gene. **h.** A second example case of AD-xQTL colocalization demonstrating three distinct CoS showing different colocalization patterns across multiple brain cell types for the *CTSH* gene. Numerical results are reported in Supplementary Data.

We applied disease-prioritized *ColocBoost* to integrate 17 xQTL data with an Alzheimer’s Disease (AD) GWAS from a recent meta-analysis study^68^ ($N_{cases}=111,326$, $N_{controls}=677,663$ including 46,828 proxy cases from UK Biobank), identifying 188 colocalization events spanning 120 distinct loci where a 95% CoS linked AD to at least one gene-level xQTL trait (Figure 6a). 61.8% of these loci were not discovered by GWAS-only fine-mapping using SuSiE at 95% credible-set (CS) coverage, suggesting that integration with molecular QTLs can uncover disease associations that are not marginally significant in GWAS. To assess their credibility, we applied *ColocBoost* to a second AD GWAS which has a smaller effective sample size especially for non-proxy AD cases^69^ ($N_{cases}=86,531$, $N_{controls}=676,386$ including 46,613 proxy cases from UK Biobank); this study differs from our primary AD meta-analysis GWAS in cohort composition, proxy case definitions, linkage disequilibrium, and phenotype ascertainment (details in Supplementary Note S.8). In this study, *ColocBoost* identified 55 CoS with xQTLs, 20 of which overlapped with the fine-mapped CS from the same GWAS. Among the remaining 35, 17 overlapped with fine-mapped CS in the larger study^68^. While this 48.5% overlap offer support for new GWAS loci *ColocBoost* can identify, it represents an underestimate of replicability given that even the larger study may still be underpowered for some loci, and that this comparison is not a strict downsample-based replication given the aforementioned differences between the two GWAS studies.

To compare with existing methods, we compiled additional sets of CoS via two pairwise approaches: by taking the union of CoS identified via applying COLOC on each xQTL with AD GWAS (hereafter referred as *COLOC*-*union*) and by performing the same pairwise analysis using *ColocBoost* (hereafter referred as *pairwise*-*ColocBoost*-*union*). *ColocBoost* recovered 96% of the events identified by COLOC-union, whereas COLOC-union reproduced only 38% of *ColocBoost* CoS (Figure 6b). Based on S-LDSC analysis, variants implicated by *ColocBoost* explained nearly twice as much AD heritability as COLOC-union, and the combination of *ColocBoost* CoS and AD fine-mapping CS explained 1.6 times more AD heritability than fine-mapping alone (Table S8). Although the overall outcome indicates that integrating xQTL data via AD-xQTL *ColocBoost* meaningfully strengthens causal AD variant and target gene discovery, we caution that LDSC heritability estimates for a single disease, like AD, can be noisy^70^. We further examined gene-proximal versus gene-distal (outside of gene TSS±10kb) colocalizations, finding that *ColocBoost* and pairwise-*ColocBoost*-union each identified more gene-distal regulatory loci (2.9-fold and 1.9-fold, respectively) than COLOC-union (Figure 6c). We further validated these CoS via ENCODE-rE2G model^34^, a predictive E2G framework integrating 1D chromatin activity and 3D chromatin contact features based on their concordance with CRISPR data across 354 biosamples from different cell types, tissues, and contexts^36,37^. A precision-recall analysis of CoS-gene maps with enhancer–gene maps showed 1.94 times higher recall for *ColocBoost* compared to COLOC-union, with minimal drop in precision (Figure 6d). A similar precision was observed when restricting the analysis to 63 unique *Colocboost* CoS not identified by AD fine-mapping. This recall enhancement is largely driven by (i) gene-distal CoS (Figure S11a) and (ii) cell-type or brain region-specific CoS (Figure S11b). While pairwise-*ColocBoost*-union also exhibited improved recall over COLOC-union, its performance remained slightly poorer than *ColocBoost* incorporating all xQTL data. We excluded HyPrColoc from this application because it identified only one colocalization event—between *BIN1* microglia eQTL and AD—when applied to all 18 traits (17 xQTLs and 1 AD GWAS). As previously illustrated (Figure 2b–i), HyPrColoc may struggle with heterogeneous signals across traits, which likely explains its limited yield in this application integrating diverse xQTL modalities and GWAS.

Out of 188 colocalization events detected by AD-xQTL *ColocBoos*t, 34% (63 events) involved only a single brain cell type, with 19 microglia specific events despite being underpowered as a relatively less common cell type in DLPFC (cell proportion <5%) (Figure 6e). This finding aligns with the recognized importance of microglia in AD pathogenesis^71–73^. Overall, 68 genes were linked by *ColocBoost* to a variant with high colocalization probability (MaxVCP>0.5; Figure 6f); 44% of them involved immune cells microglia or bulk monocyte. In comparison, 66 genes were associated with variants having MaxVCP>0.5 for pairwise-*ColocBoost*-union, whereas only 23 genes met this threshold for COLOC-union (Figure S11c,d, and Figure S12a,b for a relaxed score of MaxVCP>0.1). 38.1% of microglia or monocyte colocalizations also showed shared effects with other brain cell types (Figure 6e). We further evaluated microglia-colocalized genes against three curated microglia gene sets from Yang *et al*.^74^ (Figure S12c), and observed significant enrichment with two of them: microglia-specific IFNβ-responsive genes (EOO=2.49, p=0.001) and differentially expressed microglia genes under IFNβ treatment (EOO=2.29, $p=2\times 10^{-36}$). We also identified strong enrichment (EOO=4.96, p<0.01) with AD disease-progression genes in microglia^25^. A list of AD colocalization and their trait configurations is provided in supplementary material (Table S1, Figure S13a). We also identified several variants spanning multiple genes: for example, *rs679515* (*chr1:207577223:T:C*) colocalized with *CR2* in DLPFC bulk eQTLs, *YOD1* in oligodendrocytes, and *CR1* across oligodendrocytes and three additional bulk cortical eQTLs^75,76^ (MaxVCP=0.755; Figure S13b).

We highlight two examples of AD colocalization at the cis-regulatory loci of *BLNK* and *CTSH*, which were uniquely identified by *ColocBoost* as neither gene reached genome-wide significance of 5 × 10^−8^ in AD GWAS (p-values of 1.1 × 10^−7^ and 2.18 × 10^−7^ respectively, indication suggestive associations), which partly explains why they were missed by COLOC-union and HyPrColoc. In the first example, a single 95% CoS links *BLNK* to AD specifically in microglia (Figure 6g). Although *BLNK* is considered an AD risk gene (significantly upregulated in Aβ-treated cells^77^), a microglia-specific eQTL underlying suggestive AD association has not previously been reported^78,79^. This CoS comprises 22 variants in strong LD (purity 0.945), resulting in highest VCP of only 0.095. Therefore, a variant-level colocalization score (e.g., even at relaxed MaxVCP>0.1) would overlook this locus, whereas 95% CoS, a set-level metric including LD proxies, uncovered the microglial eQTL colocalization. This observation highlights the advantage of CoS compared to per-variant colocalization probabilities. *CTSH* offers a more complicated example where three CoS were identified. One of them colocalizes with AD GWAS in astrocytes and excitatory neurons (Figure 6h, Figure S14a) and contains two tightly correlated variants $\left(r=0.975\right)$. One variant, *rs2289702* (VCP=0.415), is located 29kb downstream of *CTSH* gene TSS, and was recently shown to reduce *CTSH* expression in luciferase assays by significantly disrupting a transcription-factor binding site (ZFP69B)^80–82^. The same CoS also encompasses an unproductive splicing QTL (u-sQTL, associated with splicing junction between Exon 3 and 4) and a pQTL in DLPFC, with effect directions suggesting a coherent cascade of decreased expression and protein abundance (Figure S14b). The other two CoS at *CTSH* did not colocalize with AD GWAS: one showed cell-type specific colocalization in excitatory neurons with links to unproductive splicing, while the other showed cell-type colocalization in astrocytes (Figure 6h, Figure S14). The low-to-moderate correlation across all three CoS (average *r*=0.53) suggest potentially independent regulatory paths, illustrating how multi-modal colocalization can unravel intricate regulatory architectures despite challenges in completely disentangling multiple correlated causal alleles as is the case of the first CoS.

## Discussion

*ColocBoost* fills a critical methodological gap in large-scale xQTL colocalization analysis as the first calibrated statistical method to handle many traits and multiple causal variants, at single-variant resolution by mapping each putative causal variant in conjunction with its LD proxies. As a multi-task variable selection framework with specialized gradient boosting updates, *ColocBoost* effectively identifies regulatory variants with shared causal effects across traits, improving sensitivity to map weaker effects in both simulations and real-world applications. As demonstrated by our large-scale brain xQTL analysis and AD GWAS integration, *ColocBoost* can generate variant level-multi-omics colocalization maps that reveal novel genetic loci underpinning complex molecular events relevant to complex diseases. Our multi-omics colocalization map is strongly supported by extensive orthogonal functional genomic data, underscoring its potential for guiding CRISPR screening and other functional follow-up studies.

Multi-trait colocalization has inherent synergies with multi-trait fine-mapping, which has been extensively studied in several past and recent research works^15,41,83^. From a broader perspective, the SEC learners employed in *ColocBoost* draw inspiration from the SER concept in SuSiE^39^, yet are fundamentally distinct from SER in regards to the weaker and partial updates regulated by a dynamic learning rate, the LD proximity smoothing, and the adaptive scheduling of SEC updates across multiple traits. Much like typical machine learning models, *ColocBoost* relies on several hyperparameters for these components. We performed exhaustive numerical assessment to confirm that our default settings robustly achieved reasonable power and *strict* FDR control across diverse scenarios (Methods, Supplementary Note S.5.2, Figure S4), while still allowing advanced users to fine-tune for specific needs. Several Bayesian multivariate frameworks based on SuSiE, including mvSuSiE^83^ for multi-trait and meSuSiE^84^/SuSiEx^85^ for multi-ancestry fine-mapping, have been proposed in different contexts of multi-trait analysis. Among them, mvSuSiE is the only approach that can effectively handle similar numbers of traits as *ColocBoost*; however, it relies on a genome-wide, empirically estimated prior in a *multivariate regression framework*, which can be powerful in homogeneous datasets but is less suitable for integrating highly diverse molecular and GWAS modalities. Although in theory one could produce colocalization-like results via mvSuSiE by estimating an overarching multi-trait prior, fine-mapping putative causal variants and assigning them to relevant traits, in practice we found this approach infeasible for large, heterogeneous xQTL and GWAS data. Specifically, mvSuSiE can struggle when priors are ill-defined, individual-level data are partially missing across modalities, or the shared LD reference mismatches certain traits. *ColocBoost*, being a *multi-task learning model*, offers greater flexibility in integrating heterogeneous traits: it avoids rigid global priors and incrementally updates colocalization signals via learners with adaptive scheduling. Although such learners reflect certain assumptions about underlying genetic architecture, they represent a more flexible framework for identifying local sharing patterns across traits not captured by global Bayesian priors. We additionally found *ColocBoost* to be more resilient to LD mismatches in summary statistics compared to mvSuSiE, owing to its LD proximity smoothing and the capability to utilize multiple trait specific LD datasets. A more detailed summary of additional methods and their limitations can be found in Table S9. Of these methods we note that CAFEH^86^, another SuSiE extension intended to perform fine-mapping and colocalization, suffers from poor calibration (high FDR)^83^, leading us to exclude it from direct comparisons in this work.

Our work has several downstream applications. First, the gradient boosting framework can potentially be repurposed for cross-ancestry GWAS fine-mapping by assembling SEC whose variant-trait configurations support cross-ancestry signals. Second, *ColocBoost* can likewise serve as a pleiotropy identification method by extending the application to multiple GWAS traits. Third, MaxVCP and CoS generated from *ColocBoost* can be used to improve functionally informed polygenic risk prediction within and across ancestries, as well as to inform functional characterization experiments, such as CRISPR^28,87^ and MPRA^88,89^. Finally, while we here showcased only 17 xQTL contexts, we have already applied *ColocBoost* in over 80 distinct molecular QTL datasets beyond eQTL, sQTL, and pQTL, and leveraged the resulting MaxVCP to general novel resources for disease-informed variant-to-function links, as reported in the FunGen-xQTL flagship manuscript^22^.

Our work has several limitations, representing important directions for future research. First, certain omics data—such as thousands of highly correlated chromatin accessibility peaks or methylation sites—can pose scalability challenges, although simple dimensionality-reduction steps^90^ or wavelet-based transformations can help^91^ (but we are yet to test these scenarios using *ColocBoost*). Second, *ColocBoost* does not currently model phenotypic or genetic correlations across traits within SEC; extending it to do so, akin to MTAG ^92^, may further improve power (Supplementary Note S.2.5). Third, the multi-task regression currently comprises of linear models, though in principle one can accommodate generalized likelihoods for count-based, binary, or mixed outcomes by developing corresponding SEC updates. Fourth, like any fine-mapping technique, *ColocBoost* assumes consistent LD reference data with summary statistics data, and thus careful QC is essential if LD reference differs substantially from the underlying cohort or alleles do not align. An LD-free implementation, mirroring HyPrColoc with single-causal assumption, is available for applications without a reliable LD panel (Figure S3f). Fifth, *ColocBoost* does not currently integrate functional annotations into SEC updates^93^. Given that functional annotations have been shown to significantly enhance fine-mapping, incorporating them into *ColocBoost* could potentially enjoy similar benefits. Finally, the application of *ColocBoost* in this work has been focused on eQTLs, sQTLs and pQTLs; as part of the FunGen-xQTL effort, we are currently expanding the application to novel types of non-coding RNA QTLs, APA QTLs, m6A QTLs, as well as exploring multi-ancestry QTL data. We envisage *ColocBoost* as a core framework for our next wave of integrative xQTL and GWAS studies.

## Methods

For L traits $Y_{1}\in {\mathbb{R}}^{N_{1}\times 1},\cdots ,Y_{L}\in {\mathbb{R}}^{N_{L}\times 1}$ and corresponding standardized (centered and scaled) genotype matrices $X_{1}\in {\mathbb{R}}^{N_{1}\times P_{1}},\cdots ,X_{L}\in {\mathbb{R}}^{N_{L}\times P_{L}}$ where $N_{l}$ is the number of individuals and $P_{l}$ is the number of genetic variants for trait l, a multiple linear regression model for genetic association can be written as $Y_{l}∼N\left(X_{l}\beta_{l},\sigma_{l}^{2}I_{N_{l}}\right)$ with fixed effect size $\beta_{l}$. In practice, we standardize $Y_{l}$ and, without loss of generality, drop $\sigma_{l}^{2}$ hereafter to lighten the notation. *ColocBoost* considers a joint loss function from L such models, $𝓛\left(\beta_{1},\cdots ,\beta_{L}\right):=\sum_{l=1}^{L}{\left\|Y_{l}-X_{l}\beta_{l}\right\|}_{2}^{2}$ and seeks to minimize $\ell_{\left(\infty ,1\right)}$ norm of its gradients by first aggregating contribution across all traits at variant j through an $\ell_{1}$-type sum, and then maximizing over j for variant with largest cross-trait gradient through an $\ell_{\infty }$-type maximum,

$${\left\|\nabla 𝓛\left(\beta_{1},\cdots ,\beta_{L}\right)\right\|}_{\left(\infty ,1\right)}=\max\left\{\sum_{l=1}^{L}\left|X_{l,j}^{T}\left(Y_{l}-X_{l}\beta_{l}\right)\right|;\forall j=1,2,\cdots ,P\right\}.$$

A standard gradient descent algorithm can be used to fit the model by maximizing the trait-specific gradient contribution to the objective,

$$g\left(r_{l}\right)=\max\left\{\left|X_{l,j}^{T}r_{l}\right|;\forall j=1,2,\cdots ,P\right\}$$

where $r_{l}=Y_{l}-X_{l}\beta_{l}$ is the residual vector at a specific boosting round (to simplify the notation, hereafter we drop the trait index l). This maximization yields the *best single-effect* variant, $j_{k}^{*}:=argmax\left|X_{j}^{T}r^{k}\right|$, with effect size updated at boosting round k through a weak learner using a dynamic learning rate, $\beta_{j_{k}^{*}}^{k+1}=\beta_{j_{k}^{*}}^{k}-\eta_{k}\mathrm{sgn}\left(X_{j_{k}^{*}}^{T}r^{k}\right)$.

### Proximity smoothing gradient boosting in genetic mapping of one trait (*FineBoost*)

Updating the effect only at the best single-effect variant $j_{k}^{*}$ identified by optimizing the objective in the previous section may be suboptimal in the presence of multiple highly correlated variants (i.e., variants in high LD), as the selected variant might not correspond to the true causal one. To account for the uncertainty introduced by LD, *ColocBoost* (referred to as *FineBoost* when L=1) employs a *proximity smoothed gradient boosting* update, where it probabilistically incorporates the LD proxies of $j_{k}^{*}$ by applying a convex relaxation to the gradient. Specifically, it replaces the naïve gradient with a smoothed version that distributes the update across the LD proximity as follows,

$$g_{smooth}\left(r^{k}\right)=\max_{\omega^{k}}{\text{Σ}}_{j}\omega_{j}^{k}\left|X_{j}^{T}r^{k}\right|/\left(N-1\right)-\tau \cdot \rho \left(\omega^{k}\right),\;\;\text{subject}\;\;\text{to}\;\;{\text{Σ}}_{j=1}^{P}\omega_{j}^{k}=1,\omega_{j}^{k}\geq 0,$$

where $\rho \left(\omega^{k}\right)={\text{Σ}}_{j=1}^{P}\omega_{j}^{k}\log\left(\omega_{j}^{k}\right)-{\text{Σ}}_{j=1}^{P}\omega_{j}^{k}\log\left(\delta_{j}^{k}\right)$ is a Kullback-Leibler divergence-based regularizer. The first term, entropy of $\omega^{k}$, encourages the update mass at $j_{k}^{*}$ to be spread more uniformly on other variants; whereas the second term, the cross-entropy with a data-driven local association simplex $\delta^{k}$, guides the mass to concentrate around LD proxies of $j_{k}^{*}$. We chose $\delta_{j}^{k}\propto \exp\left(\sqrt{\left|LD_{j,j_{k}^{*}}\right|}\times h\left(Z_{X_{j},r^{k}}\right)\right)$ where $LD_{j,j_{k}^{*}}$ denotes LD between variants j and $j_{k}^{*}$, and $h\left(Z_{X_{j},r^{k}}\right)=\left(1-\lambda \right)\left|Z_{X_{j},r^{k}}\right|+\frac{\lambda }{2}Z_{X_{j},r^{k}}^{2}$ is an elastic-net type function of the z-score testing association between $X_{j}$ and $r^{k}$. This formulation ensures the simplex stay close to $j_{k}^{*}$ while accounting for LD structure in proximity. While we assume common LD structure here for simplicity, *ColocBoost* can also accommodate trait-specific $LD_{l}$ in its formulation. We set the default value of λ to 0.5 based on extensive numerical studies. Solving the optimization over $\omega^{k}$ yields a close-form solution, leading to a smoothed extension of the sub-gradient optimization problem as follows (detailed in Supplementary Note S.1 and S.3),

$$g_{smooth}^{\tau }\left(r^{k}\right)=\tau \log\left(\sum_{j=1}^{P}\delta_{j}^{k}\exp\left(\frac{\left|X_{j}^{T}r^{k}\right|/\left(N-1\right)}{\tau }\right)\right)$$

which remains convex and differentiable with respect to $r^{k}$. The temperature parameter τ controls the degree of smoothing over g, with smaller values concentrating the update closer around $j_{k}^{*}$ (default τ=0.01). The gradient of $g_{smooth}^{\tau }\left(r^{k}\right)$.with respect to $r^{k}$ is

$$\nabla g_{smooth}^{\tau }\left(r^{k}\right)=\sum_{j=1}^{P}\xi_{j}\left(r^{k}\right)\mathrm{sgn}\left(X_{j}^{T}r^{k}\right)X_{j},$$

with

$$\xi_{j}\left(r^{k}\right):=\frac{\frac{1}{N-1}\cdot \delta_{j}^{k}\exp\left(\frac{\left|X_{j}^{T}r^{k}\right|/\left(N-1\right)}{\tau }\right)}{{\text{Σ}}_{j=1}^{P}\delta_{j}^{k}\exp\left(\frac{\left|X_{j}^{T}r^{k}\right|/\left(N-1\right)}{\tau }\right)}.$$

Subsequent updates of residual and effects using a dynamic learning rate $\eta_{k}$ yields

$$\begin{array}{l} r^{k+1}=r^{k}-\eta_{k}\nabla g_{smooth}^{\tau }\left(r^{k}\right)=r^{k}-\eta_{k}{\text{Σ}}_{j=1}^{P}\xi_{j}\left(r^{k}\right)\mathrm{sgn}\left(X_{j}^{T}r^{k}\right)X_{j},\\ \;\;\;\;\;\;\;\;\;\;\;\;\;\;\;\;\;\;\;\;\;\;\;\;\;\;\;\;\beta_{j}^{k+1}=\beta_{j}^{k}+\eta_{k}\xi_{j}\left(r^{k}\right)\mathrm{sgn}\left(X_{j}^{T}r^{k}\right), \end{array}$$

with $\eta_{k}$ adaptively tuned at each boosting round at a sufficiently small value to ensure each update behave as a *weak learner*, making controlled, incremental updates to prevent overfitting while capturing weak effects (Supplementary Note S.1).

### Coupling updates across traits to identify shared causal effects (*ColocBoost*)

*ColocBoost* introduces multi-trait weak learners called *Single-Effect Couplers* (SEC), which, at each iteration, models a single causal variant by first evaluating the coupling across traits, and then performing proximity smoothed update at the variant for each of the coupled traits. *ColocBoost* adaptively schedules boosting updates to ensure colocalization signals are progressively refined over iterations and that more accurate sets of colocalizing traits are identified at each iteration.

For multiple traits L, we define the best single-effect variant $j_{k}^{*}$ at boosting round k for *joint updates* across traits as

$$j_{k}^{*}=argmax_{j}\left(\sum_{l=1}^{L}\left|X_{l,j}^{T}\left(Y_{l}-X_{l}\beta_{l}^{k}\right)\right|\right)=argmax_{j}\left(\sum_{l=1}^{L}\left|X_{l,j}^{T}r_{l}^{k}\right|\right),$$

and the trait-specific best single-effect variant $j_{k}^{l}=argmax_{j}\left(\left|X_{l,j}^{T}r_{l}^{k}\right|\right)$ for l=1,⋯,L. We define equivalence of $j_{k}^{l}∼j_{k}^{*}$ between $j_{k}^{l}$ and $j_{k}^{*}$ if (i) $j_{k}^{l}=j_{k}^{*}$ or they are in strong LD with each other $\left(\left|r\right|>0.8\right)$), and (ii) have comparable effects, i.e., $\Delta loglik\left(\cdot ;Y_{l}\right)$ for trait l at the two variants is smaller than ε (default ε=0.1), where $\Delta loglik\left(\cdot ;Y_{l}\right)$ measures the drop in trait l ’s profile log-likelihood when effect at a given variant is set to zero. Therefore, determining which traits to update jointly reduces to evaluating whether each trait’ $j_{k}^{l}$ is *equivalent* to $j_{k}^{*}$. Let ${𝓡}_{k}^{*}$ be a subset of traits with $j_{k}^{*}∼j_{k}^{l}$ and the complementary subset ${𝓡}_{k}^{\neg *}$ with $j_{k}^{*}≁j_{k}^{l}$ at boosting round k. We propose a *dynamic coupling strategy* as follows:

#### Logic 1 (SEC).

If $j_{k}^{*}∼j_{k}^{l}$ for all $l=1,\cdots ,L\left({𝓡}_{k}^{\neg *}=\emptyset \right)$, we couple all updates across traits at the same variant $j_{k}^{*}$ with trait-specific proximity smoothing.

#### Logic 2.

If $j_{k}^{*}≁j_{k}^{l}$ for all $l=1,\cdots ,L\left({𝓡}_{k}^{*}=\emptyset \right)$, we consider the following two sub-logics to determine either trait-specific update or coupled effect update:

In *sub-logic 2.1,* if for any trait $l\in {𝓡}_{k}^{\neg *}$ there exists at least another trait $l^{\prime }$ such that $j_{k}^{l}∼j_{k}^{l^{\prime }}$, we partition traits into different equivalence groups and update *the trait group* ${𝓡}_{k}^{{*}^{\prime }}$ where maximum $\Delta loglik\left(j_{k}^{l};r_{l}^{k}\right)$ of ${𝓡}_{k}^{{*}^{\prime }}$ is the highest among all groups. To do so, we re-compute $j_{k}^{{*}^{\prime }}$ limited to ${𝓡}_{k}^{{*}^{\prime }}$, then implement *Logic 1* on ${𝓡}_{k}^{{*}^{\prime }}$ for the current boosting round.

In *sub-logic 2.2*, if there exists a subset of uncoupled traits $\left({𝓡}_{k}^{\neg {*}^{\prime }}\subseteq {𝓡}_{k}^{\neg *}\right)$, each having a unique best single-effect variant not equivalent to other traits $j_{k}^{l}∼j_{k}^{l^{\prime }}$ for any $l^{\prime }=1,\cdots ,L$), we update each trait l in ${𝓡}_{k}^{\neg {*}^{\prime }}$ at its own best $j_{k}^{l}$ and skip updates for other traits.

#### Logic 3 (delayed SEC).

If $j_{k}^{*}∼j_{k}^{l}$ for a subset of traits ${𝓡}_{k}^{*}$ and $j_{k}^{*}∼j_{k}^{l}$ for the complementary ${𝓡}_{k}^{\neg *}\left({𝓡}_{k}^{*}\neq \emptyset \;\;\text{and}\;\;{𝓡}_{k}^{\neg *}\neq \emptyset \right)$, we need to account for the possibility that some traits in ${𝓡}_{k}^{\neg *}$ do in fact share causal effect at $j_{k}^{*}$ but currently fail the *equivalence* check because their best single-effect update is another stronger, trait specific effect at a different variant. We ensure this by avoiding premature coupled updates based only on ${𝓡}_{k}^{*}$, through a *delayed SEC* (D-SEC) approach to update ${𝓡}_{k}^{\neg *}$ at current boosting round, deferring updates at $j_{k}^{*}$ into future rounds. To implement D-SEC:

*In*| *sub-logic 3.1*, if $\min_{l\in {𝓡}_{k}^{*}}\left\{\Delta loglik\left(j_{k}^{*};r_{l}^{k}\right)\right\}>\max_{l\in {𝓡}_{k}^{\neg *}}\left\{\Delta loglik\left(j_{k}^{l};r_{l}^{k}\right)\right\}$, we identify ${𝓡}_{k}^{\neg {*}^{\prime }}\left({𝓡}_{k}^{\neg {*}^{\prime }}\subseteq {𝓡}_{k}^{\neg *}\right)$ whose Δloglik at $j_{k}^{*}$ is substantial (above given threshold, Supplementary Note S.2.3), and implement *Logic 2 on* ${𝓡}_{k}^{\neg {*}^{\prime }}$. If no such additional traits exist, we implement *Logic 1* on ${𝓡}_{k}^{*}$ at variant $j_{k}^{*}$.

*In sub-logic 3.2*, if $\min_{l\in {𝓡}_{k}^{*}}\left\{\Delta loglik\left(j_{k}^{*};r_{l}^{k}\right)\right\}<\max_{l\in {𝓡}_{k}^{\neg *}}\left\{\Delta loglik\left(j_{k}^{l};r_{l}^{k}\right)\right\}$, we skip any updates at $j_{k}^{*}$, instead implement *Logic 2* on ${𝓡}_{k}^{\neg *}$.

Detailed evaluation on the proposed dynamic coupling strategy is provided in Supplementary Note S.2, including a simplified version of L=2 traits as a special case to help readers more easily understand the strategy in a pairwise colocalization setting.

### Expediated boosting learners for disease-prioritized *ColocBoost*

We introduce an *expedited SEC* (E-SEC) to prioritize identifying colocalization with a focal trait, e.g., a GWAS, implementing the disease-prioritized mode of *ColocBoost*. We start with the best single-effect variant for GWAS of interest $j_{k}^{e}=argmax_{j}\left(\left|X_{e,j}^{T}r_{e}^{k}\right|\right)$, and construct its equivalence group ${𝓡}_{k}^{e}$ with respect to $j_{k}^{l}=argmax_{j}\left(\left|X_{l,j}^{T}r_{l}^{k}\right|\right)$ for each trait l≠e by checking if $j_{k}^{e}∼j_{k}^{l}$. If ${𝓡}_{k}^{e}\neq \emptyset$, we re-compute $j_{k}^{*}=argmax_{j}\left({\text{Σ}}_{l\in {𝓡}_{k}^{e}}\left|X_{l,j}^{T}r_{l}^{k}\right|\right)$ across traits in ${𝓡}_{k}^{e}$ as the candidate variant to update in current boosting round, where by design, $j_{k}^{e}∼j_{k}^{*}$. We then employ the dynamic coupling strategy previously described to guide subsequent boosting rounds. This procedure is iterated until ${𝓡}_{k}^{e}=\emptyset$. In doing so, traits in ${𝓡}_{k}^{e}$ are prioritized for coupling at variant $j_{k}^{*}$ during earlier rounds, thereby improving detection of colocalizations with the focal trait.

### Inferences on colocalization events

Each colocalization event s identified by *ColocBoost* corresponds to a triplet $\left\{CoS_{s}\left(\alpha \right),T\left(s\right),g\left(s\right)\right\}$, where the trait configuration, $T\left(s\right)$ can also be analogously represented as a configuration vector $T^{s}=\left(T_{1}^{s},\cdots ,T_{L}^{s}\right)$, where $T_{l}^{s}\in \left\{0,1\right\}$ indicates whether the variant has a putative effect on trait l. For any given variant, the total number of possible configurations across L traits is $2^{L}-L-1$. Let ${𝓚}_{s}$ be the subset of boosting rounds corresponding to configuration $T^{s}$. For each trait l with $T_{l}^{s}=1$, we define ${𝓦}_{l}^{s}$ as the collection of weight vectors $\xi_{l}^{k}=\left\{\xi_{jl}^{k}\right\}$, where $\xi_{jl}^{k}$ denotes the weight update of variant j at round $k\in {𝓚}_{s}$ as previously introduced in the section describing *FineBoost*. By virtue of being weak learners, different SEC may update the same or nearby (LD-proximal) variants over multiple rounds for the same causal effect. This often yields similar weight vectors $\xi^{k}$ and ${\xi^{k}}^{\prime }$, which, by design, further enhances accommodation of inherent signal uncertainly in high-LD regions. To assemble these SECs, we aggregate $\xi_{l}^{k}$ for $k\in {𝓚}_{s}$ using a modularity-based hierarchical clustering approach (Supplementary Note S.4). Then, within each cluster, we re-normalized the per-round weights $\xi_{jl}^{k}$ by their corresponding profile log-likelihood gains $\Delta 𝓛\left(k+1,k\right)=𝓛\left(\beta_{1}^{k+1},\cdots ,\beta_{L}^{k+1}\right)-𝓛\left(\beta_{1}^{k},\cdots ,\beta_{L}^{k}\right)$. This yields a variant-level probability of effect under $T^{s}$ for trait l,

$$W_{jl}^{s}=\frac{{\text{Σ}}_{k\in {𝓚}_{s}}\left|\Delta 𝓛\left(k+1,k\right)\right|\times \xi_{jl}^{k}}{{\text{Σ}}_{k\in {𝓚}_{s}}\left|\Delta 𝓛\left(k+1,k\right)\right|}$$

and $W_{j}^{s}:={\prod_{l\in C^{s}}\left(W_{jl}^{s}\right)}^{v/\left|T^{s}=1\right|}$ which aggregates all traits with $T_{l}^{s}=1$ into variant-level probability for variant j supporting $T^{s}$. We set v=1.5 by default to ensure consistent scale across different numbers of colocalizing traits. As previously described (in the Overview of Methods), $W_{j}^{s}$ can be used to construct α-level CoS (colocalization confidence sets) and variant colocalization probabilities (VCP),

$$\begin{array}{l} CoS_{s}\left(\alpha \right)=\left\{j_{1},\cdots ,j_{p_{0}}:p_{0}=\min\left(p:\sum_{j=1}^{p}W_{\left(j\right)}^{s}\geq \alpha \;\;\;;\;\;\;W_{\left(1\right)}^{s}\geq \cdots \geq W_{\left(P\right)}^{s}\right)\right\},\\ \;\;\;\;\;\;\;\;\;\;\;\;\;\;\;\;\;\;\;\;\;\;\;\;\;\;\;\;\;\;\;\;\;VCP_{j}=1-\prod_{s=1}^{S}\left(1-W_{j}^{s}\right). \end{array}$$

Similar to SuSiE^39^, we discard the CoS with low *purity* (minimum absolute correlation between all pairs of variants within CoS, default to purity < 0.5) and with small $\Delta {𝓛}_{1}$ and $\Delta {𝓛}_{l}^{s}$, the relative contribution to the trait-specific profile log-likelihood and CoS-trait-specific log-likelihood respectively ($\Delta {𝓛}_{l}<0.025$ and $\Delta {𝓛}_{l^{s}}<0.1\Delta {𝓛}_{1}$ by default). We also introduced strategies to further handle highly correlated CoS, to diagnose quality of CoS due to incomplete LD and/or mismatch with summary statistics, and to filter potentially spurious signals (Supplementary Note S.4).

### Summary statistics extension and related practical features

We provide implementation details in the Supplementary Note S.2.5 describing how *ColocBoost* addresses common challenges in real-world genetic data integration. First, *ColocBoost* supports both individual-level and summary-statistics-based analyses, with optional use of multiple external LD reference panels when individual-level data are unavailable. Second, it accommodates mixed data types across traits, as in our GWAS–xQTL integration. Third, it handles non-overlapping variant sets without requiring imputation or variant exclusion while retaining variants with partially missing LD data. Finally, it provides an LD-free mode under a single causal variant assumption, enabling analysis when LD is unavailable or poorly matched.

### Simulation settings for multi-trait colocalization methods performance benchmark

To evaluate multi-trait colocalization methods, we used genotype data from the ROSMAP cohort $\left(N=1,162\right)$ on 1,287 gene regions (±1.5Mb from gene TSS) randomly drawn from topologically associating domains (TAD) across the genome^43^. Each region contains variants filtered by missing rate less than 0.1 and minor allele frequency (MAF) greater than 0.05, resulting in between 2,069 to 40,000 variants per region (average 9,216). Let X be the standardized genotype matrix in each region and $\beta_{l}$ be the effect sizes for variants on trait l, where $P_{c}$ approximate independent variants were sampled to have causal (non-zero) effects and all the remaining were set to non-causal (zero effect). We simulate the normalized effect sizes such that the percentage of phenotypic variance explained (PVE) by each variant $\phi =Var\left(X_{j}\beta_{i,j_{1}}\right)/Var\left(Y_{l}\right)$ for $j_{1},\cdots ,j_{P_{c}}$ is kept fixed. Then, we generated simulated traits based on $Y_{l}=X\beta_{l}+\epsilon$ with $\epsilon ∼𝓝\left(0,\sigma_{y}^{2}I\right)$, where $\sigma_{y}^{2}$ is chosen such that a modest ϕ=0.05 was used for the xQTL-only colocalization setting, and a lower ϕ=0.02 use for disease-prioritized setting. While this ϕ may appear relatively large for some GWAS traits, real GWAS typically benefit from much larger sample sizes to detect weaker effects with comparable power. Our choice of ϕ for simulation purpose is merely a practical trade-off given limited availability to large-scale genome sequence data. For under the null, we set $\beta_{l}=0$ and vary $\sigma_{y}^{2}$ from 0.1 to 1. To simulate dependency between traits due to non-genetic factors such as sample overlap, we model residual as $\epsilon ∼𝓝\left(0,\sigma_{y}^{2}\text{Σ}\right)$, where Σ was estimated from FunGen-xQTL atlas used in our real data analysis (Supplementary Note S.6).

Our primary numerical study considered three broad scenarios based on different number of traits simulated: (i) For simulations with only two traits, we assumed both traits shared the same $P_{c}$ causal variants, where $P_{c}$ varied from 1 to 3. (ii) For simulations with 5,10, and 20 traits, we adopted a more complex simulation design to mimic the number of causal variants and variant-trait configurations in real world data analyses. Specifically, we varied the number of causal variants, ranging from 1 to 5, and randomly assigned each variant to subset of traits to determine the colocalization patterns at each gene locus based on an empirical probability distribution derived from the observed genome-wide overlap of fine-mapping credible sets in FunGen-xQTL fine-mapped atlas^22^ (Table S10). For example, when simulating 3 causal variants per gene across 10 traits, we randomly selected 2–10 colocalized traits for each causal variant with probabilities 38.0%, 28.5%, 16.3%, 7.9%, 5.1%, 2.4%, 1.2%, 0.4%, °0.2%. (iii) For simulations with 50 traits, we also varied the number of causal variants, ranging from 1 to 5, and randomly selected 10–25 colocalized traits for each causal variant. Notably, as the number of traits increases, the colocalization patterns tend to become sparser. In some cases, distinct subsets of traits may share different causal variants (i.e., traits $\left\{1,2,3\right\}$ shared one causal variant, while traits $\left\{4,5,6\right\}$ shared another one). In addition, we conducted four other simulation experiments each featuring simpler colocalization patterns drawn from published work describing competing methods, whose design are briefly described in Results and detailed in Supplementary Note S.6.

### Performance metrics for benchmarking multi-trait colocalization methods

We used two principal metrics to compare *ColocBoost* with other methods: (i) statistical power and (ii) false discovery rate (FDR), evaluated at the level of a colocalization configuration, for both the 95% CoS and the set of traits involved in this configuration. We define *power* as the proportion of correctly identified CoS-trait pairs over the total number of true variant-trait pairs in simulated causal configurations. Specifically, let ${𝓣}_{j,true}$ be the set of true colocalized traits for the true causal variant j for $j\in {𝓜}_{causal}$, where ${𝓜}_{causal}$ is the set of true causal variants. Let $CoS_{s}$ denote the estimated 95% CoS for colocalization event s and ${\hat{𝓣}}_{s}$ be the set of colocalizing traits associated with this event. For each true causal variant j, *power* is calculated as

$$p_{j}=\left\{\underset{0;}{\max\left(\left|{𝓣}_{j,true}\cap {\hat{𝓣}}_{s}\right|\right);}\right.\;\;\;\;\;\;\;\underset{\text{otherwise},}{\text{if}\;\exists s\;\;\text{s}.\text{t}.j\in CoS_{s}\;\;and\;\;{\hat{𝓣}}_{s}\subset {𝓣}_{j,true},}$$

$$\text{Power}=\frac{{\text{Σ}}_{j\in {𝓜}_{causal}}p_{j}}{{\text{Σ}}_{j\in {𝓜}_{causal}}\left|{𝓣}_{j,true}\right|}.$$

where the notation $\left|𝓣\right|$ denotes the number of elements in the set 𝓣. This definition provides a stringent measure of detection accuracy: it ensures that the detected colocalized traits must be a subset of the true set for that variant, and among such cases, power is proportional to the number of correctly identified traits.

We defined FDR as the proportion of incorrectly detected CoS over all detected CoS. Specifically, for each detected $CoS_{s}$ and colocalized traits ${\hat{𝓣}}_{s}$, we considered two stringent conditions to classify false discoveries: (i) $CoS_{s}$ does not include the true causal variant and (ii) $CoS_{s}$ includes a causal variant j, but also includes incorrect traits $\left({\hat{𝓣}}_{s}⊄{𝓣}_{j,true}\right)$. We assigned $F_{s}=1$ if either of these two conditions was met and $F_{s}=0$ otherwise. The FDR is calculated as

$$FDR=\frac{{\text{Σ}}_{s=1}^{S}F_{s}}{S}.$$

We further evaluate *variant-level* detection accuracy using *precision-recall curves (PRC)*, a standard practice adopted in a similar context (fine-mapping) when variant-level scores are determined by both signal strength and its LD proximity and is difficult to separate apart these two factors^39,84,94–97^. In brief, we applied a range of thresholds $\left[0,1\right]$ to each variant’s colocalization score to compute precision and recall rates. For *ColocBoost*, we used VCP as the colocalization score; see Supplementary Note S.6 for details of colocalization score of other methods.

### Comparison with OPERA using region-level benchmarks

We next compared *ColocBoost* with the recently proposed multi-omics colocalization method OPERA by simulating data at the region level for 2, 5, and 10 traits. In each locus, the first trait served as the *target* trait with per-variant PVE, ϕ, ranging from 0.02 to 0.05, while the remaining traits each had ϕ=0.05. The number of causal variants and colocalizing traits per variant were randomly assigned based on distribution drawn from genome-wide overlaps of fine-mapped credible sets previously described. Because OPERA requires genome-wide GWAS summary statistics for estimating its hyperparameters, we computed summary statistics for 500 of the 1,287 independent, non-overlapping gene regions previously described, to serve as 500 replicates for power and FDR calculations (Supplementary Note S.6).

Unlike *ColocBoost* and other methods which identifies variant-level (VCP) and locus-level (CoS) colocalizations, OPERA only provides gene-level test for whether a colocalized signal exists within an entire gene, without pinpointing the specific variant(s). OPERA does not perform xQTL-only colocalization because it requires an explicit target trait (e.g. GWAS). To enable a fair comparison between OPERA and *ColocBoost*, we consider the GWAS-prioritized mode of *ColocBoost*, and defined power and FDR at gene-level evaluated on the target trait. Specifically, we defined power as the proportion of correctly identified gene regions colocalized with the target trait over the total true colocalized gene regions. FDR is defined as the proportion of incorrectly identified gene regions colocalized with the target trait over all identified regions. In practice, OPERA may report multiple overlapping subsets ambiguous as to which true colocalization they correspond to due to a lack of variant level information. For instance, for cases with two distinct colocalizations $\left\{1,2\right\}$ an d $\left\{1,2,3\right\}$ where trait 1 is the target trait, OPERA often reports three subsets $\left\{1,2,3\right\}$, $\left\{1,2\right\}$ and $\left\{1,3\right\}$, the last of which is ambiguous as to whether it is a false positive driven by a incorrect causal variant, or merely subset of $\left\{1,2,3\right\}$ with the correct underlying causal variant. We adopted a lenient evaluation strategy for OPERA: for both the ground truth and reported colocalization, we merged any subsets that are identical or strictly nested into their largest compatible set (here, $\left\{1,2,3\right\}$) to enable a consistent gene-level assessment. To compare on unified gene-level basis, we likewise apply the same merging strategy to *ColocBoost* CoS, despite it often identifies multiple distinct and correct colocalization events per gene without merging. This procedure ensures a fair comparison for OPERA at gene-level despite underestimating the power of *ColocBoost* at event-level and overlooking finer colocalization patterns it can capture.

### Unified variant-level benchmarking for other competing colocalization methods

Because many existing colocalization methods report only a single top-ranked variant, we observed inflated FDRs, particularly under multiple causal variants scenarios, due to failure to account for LD-driven uncertainty in variant selection (Figure S2e). In contrast, our proposed colocalization confidence set (CoS) framework offers a more informative and robust summary (Figure 2c). However, since CoS is unique to *ColocBoost* and not natively supported by competing methods, we developed a unified evaluation approach by extending each method to construct 95% credible sets analogous to our 95% CoS. These were built using reported variant-level probabilities of each method (detailed below) and filtered with the same purity threshold (requiring purity>0.5) to ensure fair and interpretable comparisons. The construction of 95% CoS analogs for each method is as follows:

- COLOC (V5): for colocalization configuration where PP.H4 is greater than other PPs (PP.H0, PP.H1, PP.H2, PP.H3), we use ‘SNP.PP.H4’ as variant-level scores to define 95% colocalization credible set similar to how we defined 95% CoS. Unlike many analyses that apply a high PP.H4 cutoff (e.g., 0.7–0.8), we do not impose one here for methods comparison purposes, as the COLOC paper does not rigorously recommend a particular threshold, and our simulations show that omitting it preserves well-controlled FDR while maintaining power comparable to *ColocBoost*. Nonetheless, users requiring more stringent evidence can adopt a higher PP.H4 cutoff in practice.
- HyPrColoc: for identified colocalizations with posterior probability higher than uncolocalized configurations, we used ‘snp.scores’ as the variant-level scores to define 95% colocalization credible set. As with COLOC, we omit an explicit configuration probability cutoff here for fair comparison.
- MOLOC: for colocalization configuration where PPA.ab is greater than other PPAs (PPA.a, PPA.b, PPA.a,b, and zero), we used ‘SNP.PP.ab’ as the variant-level scores to define 95% colocalization credible set.

### Multi-omics and GWAS data sources and preprocessing

In our primary analyses, we used 17 *cis*-xQTL contexts spanning 16,928 genes and 3 modalities (gene expression, splicing, protein abundance) from the aging brain cortex of ROSMAP donors (average N=595; Table 2)^22,24,44–46^. Bulk RNA-seq were generated from postmortem tissues across the dorsolateral prefrontal cortex (DLPFC), posterior cingulate cortex (PCC), and head of caudate nucleus (AC). Bulk monocytes were isolated from peripheral blood mononuclear cells through negative selection, followed by fluorescent antibody labeling and sorting of live CD14+CD16- cells for downstream analysis^98^. Single-nucleus RNA-seq was performed on 479 DLPFC samples and we focused on six major cell types with abundant cell proportions to robustly perform eQTL calling: excitatory neurons (Exc), inhibitory neurons (Inh), microglia (Mic), oligodendrocyte precursor cells (OPC), oligodendrocytes (Oli), and astrocytes (Ast)^44^. For bulk RNA-seq, standard QC and adapter trimming was performed on raw reads, followed by alignment via STAR and gene expression quantification via RNASeqQC^22^. Genes were removed if over 20% samples have TPM expression level of 10% or less. Quantile normalization was applied to gene-level expression. Additionally, LeafCutter2 was applied to generate productive and unproductive splicing events for sQTL analysis, as described in another FunGen-xQTL companion project^47^. For snRNA-seq data, gene expressions were summed into pseudo bulk counts for each donor-cell pair, quantified as counts per million (CPM) and then quantile normalized. SRM proteomics was performed on DLPFC with manual inspection to ensure correct peak assignment and peak boundaries, and then the peptide relative abundances were log2 transformed and centered at the median. For all normalized molecular phenotypes, we adjusted the technical factors (batch, RNA integrity number, post-mortem interval), biological covariates (sex, age at death), the top 15 genotype PC, and additional hidden confounders using PCA on each normalized phenotype matrix with the number of PCs determined by the Marchenko-Pastur limit.

In GTEx eQTLs colocalization analysis, normalized gene expression for 17,834 genes from all samples across 13 GTEx brain tissues and genotype data were downloaded from the open access dataset from public release GTEx Analysis V8^6^ (average N=168; Table S3). Following from recommendations of GTEx consortium we adjusted 68 covariates on both normalized gene expression and genotype, including top 5 genotyping PCs, 60 PEER factors, sequencing platform and protocol, and sex.

For AD-xQTL colocalization, we constructed a custom LD reference panel from the Alzheimer’s Disease Sequencing Project (ADSP), consisting of approximately 17,000 whole-genome sequenced (WGS) individuals of European ancestry. We primarily used a recent AD GWAS meta-analysis summary statistics^68^ ($N_{cases}=111,326$, $N_{controls}=677,663$) after performing quality control against this reference panel, including allele harmonization and LD mismatch detection using SLALOM^99^, with flagged suspicious variants removed. As a secondary AD GWAS, we considered meta-analysis summary statistics with a smaller effective sample size especially for non-proxy AD cases^69^ ($N_{cases}=86,531$, $N_{controls}=676,386$ including 46,613 proxy cases from UK Biobank) (Supplementary Note S.8).

### Excess of overlap (EOO) analysis for variant-gene links

We quantify the concordance between two sets of variant-gene (VG) links using an *excess of overlap (EOO) statistic*^52^, computed by comparing the fraction of shared VG pairs to their expected overlap by chance. Specifically, for sets $VG_{1}$ and $VG_{2}$ of sizes $\left|VG_{1}\right|$ and $\left|VG_{2}\right|$ out of a total tested $T_{1}$ and $T_{2}$ VG pairs, with $T_{12}$ pairs commonly tested by both methods, we define:

$$EOO\left(VG_{1},VG_{2}\right)=\frac{\left|VG_{1}\cap VG_{2}\right|/T_{12}}{\left(\left|VG_{1}\right|/T_{1}\right)\left(\left|VG_{2}\right|/T_{2}\right)},$$

so that values above 1 indicate more overlap than expected by chance. Standard error of EOO can be estimated by leave-one-chromosome-out strategy. Analogously, we define discordance as the fraction of variants linked to different genes between two sets, divided by the amount of discordance expected by chance.

In evaluating element-gene maps from CRISPR or promoter-capture datasets, we adopt different definitions of T and VG tailored to specific tasks. We defined VG for element-gene maps as all pairs of variant-gene links with the element of interest. For variant-gene pairs across the genome (total 360,092,003), VG is defined by the 95% CoS-gene links from 95% CoS-gene links for *ColocBoost*, SNP-Gene links of marginal QTL associations, and fine-mapped eQTL 95% credible set-gene links. For the cell-type-specific analyses, we focus on 95% CoS-gene links (433,927 identified), and VG is defined to by the variant-gene pair for the corresponding cell-type.

### MaxVCP-based variant annotation and heritability analysis

To evaluate whether variants implicated by multi-trait colocalization inform disease risk, we constructed a new variant annotation based on the *maximum variant colocalization probability* (MaxVCP). Specifically, for variants in 95% CoS obtained by *ColocBoost*, let ${𝓖}_{j}$ denote the set of genes whose 95% CoS contains variant j, we then define

$$MaxVCP_{j}=\underset{\text{g}\in {𝓖}_{j}}{\max}VCP_{j}^{g},$$

where $VCP_{j}^{g}$ is the variant colocalization probability for variant j and gene g. Using ROSMAP and GTEx data we created five distinct MaxVCP based annotations as previously described in Results, for 9,991,229 variants with minor allele count ≥5from 1000 Genomes Project Europeans (Table S1). We then applied S-LDSC to quantify how well these annotations capture disease heritability across 57 GWAS summary statistics data-sets (Table S6), conditioning on 97 functional annotations from the baseline-LD v2.2 model^57^. We consider two metrics, heritability enrichment and standardized effect size $\tau^{*}$. We meta-analyze these metrics across all 57 traits, as well as separately across the blood and brain-related traits.

Enrichment is defined as the relative contribution of heritability explained by colocalized annotation (MaxVCP),

$$Enrichment=\frac{\tau_{MaxVCP}}{h_{g}^{2}/M},$$

where $\tau_{MaxVCP}=h_{g,MaxVCP}^{2}/{\text{Σ}}_{j}a_{j,MaxVCP}$ quantifies heritability explained by per-variant within colocalized annotation. Denote $h_{g}^{2}={\text{Σ}}_{j}Var\left(\beta_{j}\right)$ as the estimated variant heritability across all annotations, then $h_{g}^{2}/M$ represents the proportion of heritability explained by per-variant within all annotations, where M is the total number of variants and $Var\left(\beta_{j}\right)$ is the per-variant heritability of variant j. Standardized effect size $\left(\tau^{*}\right)$ is defined as the proportionate change in per-variant heritability associated with a one standard deviation increase in the value of the colocalized annotation, conditional on other annotations included in the model. Both enrichment and $\tau_{MaxVCP}^{*}$ are estimated via a genomic block-jackknife procedure with 200 blocks that also provides standard errors. We considered two versions of $\tau_{MaxVCP}^{*}$: a marginal version conditional on baseline-LD annotations only, and a joint version where the conditioning set includes other colocalization-derived annotations in addition to baseline-LD. More detailed description of metrics can be found in Supplementary Note S.7.

### Benchmarking AD-colocalized CoS-gene links against ENCODE-rE2G regulatory annotations

We benchmarked AD-colocalized 95% CoS-gene links from *ColocBoost* and COLOC (V5) against element-gene links in the ENCODE-rE2G model, using precision and recall to assess concordance. *Recall* was defined as the fraction of high-confidence element-gene links—*i.e.*, distinct element-gene pairs across all biosamples harboring AD GWAS loci—captured by each colocalization method. To define distinct regulatory elements, overlapping links across biosamples were merged using *bedtools*, and we retained those elements containing at least one AD GWAS variant with p-value < 10^−5^(known as the “suggestive” GWAS significance threshold). *Precision* was defined as the fraction of 95% CoS-gene links from each method that overlapped with ENCODE-rE2G element-gene links.

## Supplementary Material

Supplement 1

Supplement 2

Supplement 3

## Figures and Tables

**Table 1.** Methodological advantages of *ColocBoost* over existing colocalization approaches. A comparison of *ColocBoost* with four other colocalization methods — COLOC (V5), MOLOC, HyPrColoc and OPERA — focusing on (i) their capacity for multiple causal variants, (ii) support to diverse data-type models, (iii) ability to capture weak, distal xQTL effects, (iv) runtimes and (v) software implementation. Runtimes were assessed for a single simulated locus using a sample size of N=1,160 and P≈10K variants. For methods based on the summary statistics, the time required to generate them from individual-level simulated data were not included.

Method		*ColocBoost*	HyPrColoc	MOLOC	COLOC (V5)	OPERA
Upper limit on number of causal variants		no limit	1	1	pre-specified	-
Data accepted	Summary statistics	yes	yes	yes	yes	yes
	Individual level data					
		yes	no	no	yes*	no
	Mixed					
		yes	no	no	no	no
CoS		yes	yes^#^	yes^#^	yes^#^	-
Low effect colocalization		yes	no	no	no	no
Runtimes	2 traits	~12s	<1s	<1s	<30s	<1min
	5 traits	~30s	<1s	>10min	-	<3min
	50 traits	~4.5min	<1s	-	-	-
Software		R	R/Rcpp	R	R	command-line

# indicates that colocalization confidence/credible sets (CoS) were not provided in the original methods but can be computed using available outputs (Methods);

* COLOC(V5) only supports summary statistics by default although as a two-stage approach it can use SuSiE to process individual-level data, followed by colocalization analysis;

- indicates that the method was not applicable to the specified scenario due to its intrinsic limitations.

**Table 2.** Overview of 17 *cis*-xQTL datasets from ROSMAP brain cortex. Each row lists the molecular trait modalities and GWAS, abbreviated dataset name, cell types or brain regions corresponding to the xQTL data, and sample sizes analyzed for each dataset.

Categories	Contexts	Sample size	# genes
Pseudo-bulk eQTL* (snRNA-seq)	Microglia* (Mic) Astrocytes* (Ast) Oligodendrocytes* (Oli) Excitatory neuron* (Exc) Inhibitory neuron* (Inh) Oligodendrocyte Progenitor Cells* (OPC)	N=419	7,357 11,816 11,311 11,522 11,685 8,019
Bulk eQTL*	Dorsolateral Prefrontal Cortex* (DLPFC) Anterior Cingulate Cortex* (AC) Posterior Cingulate Cortex* (PCC) Monocyte*	N=784 N=593 N=441 N=226	16,901 16,687 16,699 12,829
Bulk sQTL	Dorsolateral Prefrontal Cortex (Productive, Unproductive)	N=806	12,613
	Anterior Cingulate Cortex (Productive, Unproductive)	N=603	12,906
	Posterior Cingulate Cortex (Productive, Unproductive)	N=449	12,976
Bulk pQTL	Dorsolateral Prefrontal Cortex	N=416	7,641
AD GWAS	AD GWAS summary statistics from Bellenguez et al. (2022)	N=788,989	16,928
	AD GWAS summary statistics from Wightman et al. (2021)	N=762,917	

Asterisks (*) mark modalities included in eQTL specific colocalization analysis as an ablation study.

## Data Availability

Full expression, splicing and protein abundance xQTL summary statistics are available to the reviewers through the following Synapse link. The complete xQTL summary statistics for the 17 brain xQTL molecular contexts will be available at the time of publication via The National Institute on Aging Genetics of Alzheimer’s Disease Data Storage Site (NIAGADS) at https://adsp.niagads.org/. The download information of full colocalization results from the xQTL-only and GWAS-xQTL *ColocBoost* can be found in Supplementary Table 1, including the CoS and VCP scores.
