# Supplementary material for: Integrative multi-omics QTL colocalization maps regulatory architecture in aging human brain
Source: medRxiv. 2025 May 6:2025.04.17.25326042. Preprint. [Version 2] doi: 10.1101/2025.04.17.25326042 (PMC12083576; doi:10.1101/2025.04.17.25326042)
Supplement: Supplement 2 [file media-2.docx]

**Integrative multi-omics QTL colocalization maps regulatory architecture in aging human brain**

Xuewei Cao^1,2^, Haochen Sun^1,2^, Ru Feng^1^, Rahul Mazumder^3,4^, Carlos F Buen Abad Najar^5^, Yang I. Li^5,6,7^, Philip L. de Jager^8,9,10^, David Bennett^11^, The Alzheimer’s Disease Functional Genomics Consortium, Kushal K. Dey^2,12,13,*^, Gao Wang^1,8,*^

^1^ Center for Statistical Genetics, The Gertrude H. Sergievsky Center, Columbia University, New York, NY, USA

^2^ Computational and Systems Biology, Sloan Kettering Institute, Memorial Sloan Kettering Cancer Center, New York, NY, USA

^3^ Operations Research Center, Massachusetts Institute of Technology, Cambridge, MA, USA

^4^ Sloan School of Management, Massachusetts Institute of Technology, Cambridge, MA, USA

^5^ Section of Genetic Medicine, University of Chicago, Chicago, IL, USA

^6^ Department of Human Genetics, University of Chicago, Chicago, IL, USA

^7^ Chan Zuckerberg Chicago, Chicago, IL, USA

^8^ Department of Neurology, Columbia University, New York, NY, USA

^9^ Center for Translational & Computational Neuroimmunology, Columbia University, New York, NY, USA

^10^ Taub Institute for Research on Alzheimer’s Disease and the Aging Brain, Columbia University, New York, NY, USA

^11^ Rush Alzheimer’s Disease Center and Department of Neurological Sciences, Rush University Medical Center, Chicago, IL

^12^ Physiology, Biophysics and Systems Biology, Weill Cornell Medicine, New York, NY, USA

^13^ Gerstner Sloan Kettering Graduate School of Biomedical Sciences, New York, NY, USA

^*^ Correspondence: Gao Wang, wang.gao@columbia.edu; Kushal K. Dey, deyk@mskcc.org

**Figure S1. Computational framework of *ColocBoost* for multi-trait colocalization analysis.** **a**. Comparison between standard gradient boosting algorithm and proximity smoothing gradient boosting algorithm in *ColocBoost*. Standard gradient boosting updates one variant in each iteration based on the strongest association of the genotypic profile of variants with the residual phenotype. Proximity smoothing gradient boosting updates this candidate best variant as well as some of its strong LD neighbors through an adaptive smoothing, determined by the proximal LD and the strength of the signal. **b**. Schematic representations of multi-trait weak learner, Single-Effect Couplers (SEC), across two traits that models a single causal variant by first evaluating the coupling across traits and then performing proximity smoothed updates at the variant for each of the coupled traits. **c**. Delayed-SEC (D-SEC) updates across two traits are designed to remove or substantially weaken uncoupled signals before performing coupled updates. **d**. Comprehensive workflow diagram depicting the integration of SEC and D-SEC within an iterative gradient boosting informed algorithm in *ColocBoost* for multi-trait colocalization analysis.


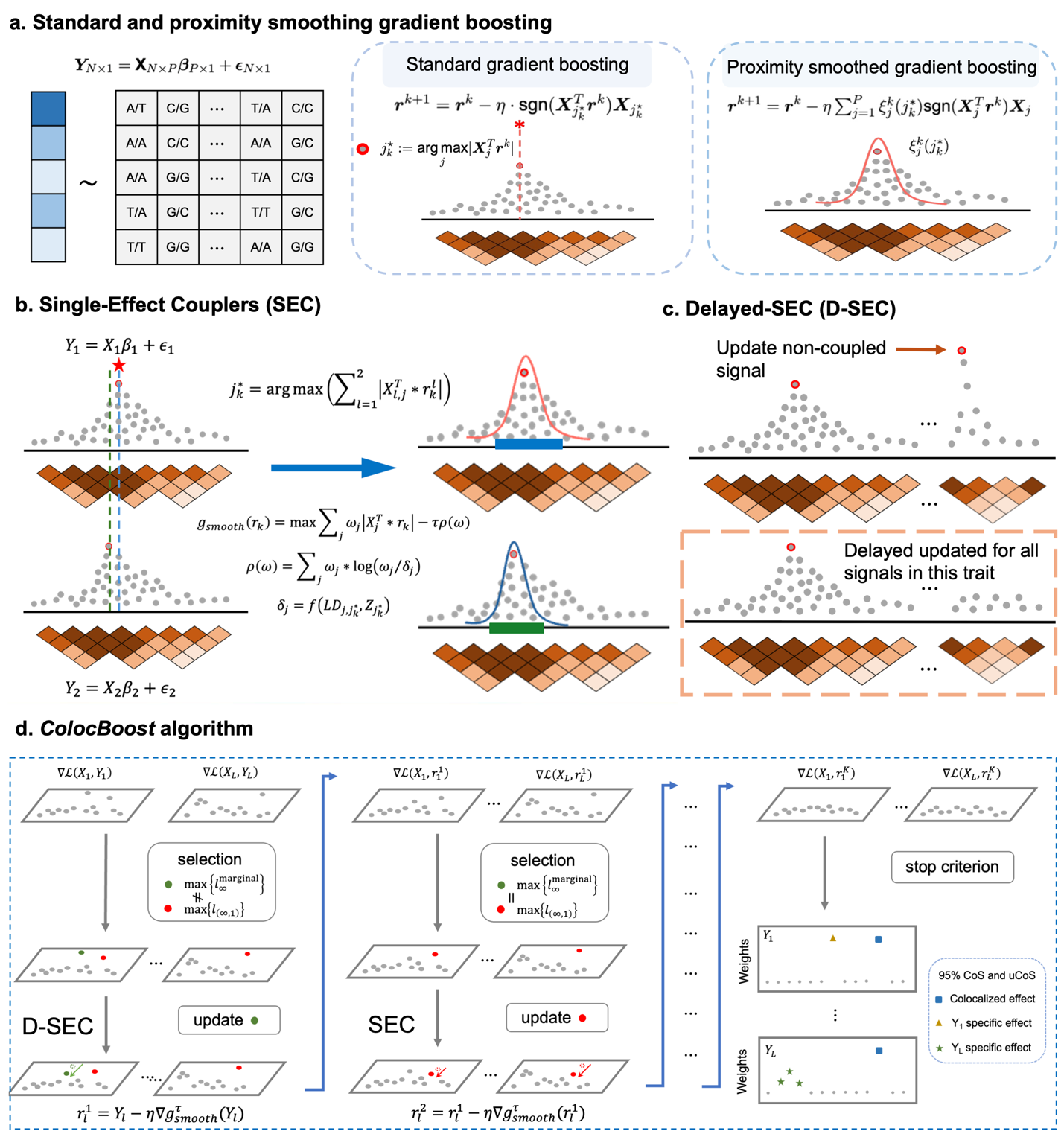


**Figure S2. Extended performance comparison of *ColocBoost* against other multi-trait colocalization methods. a.** Schematics of a broad range of simulation designs used for comprehensive benchmarking, including realistic simulation analyses, simulation designs adopted in original publications of competing methods, and simulation with correlated traits. (See details in **Supplementary Note**). **b.** Scatter plot of the negative logarithm of False Discovery Rate (-log(FDR)) and power of *ColocBoost* and HyPrColoc across varying numbers of true causal variants (see **Methods** for details on the computation of power and FDR). The vertical red line represents the FDR cut-off of 0.5. **c.** Size of CoS and purity comparisons in simulation designs involving 2, 5, 10, and 20 traits. **d.** Statistical power and FDR of *ColocBoost* and HyPrColoc in simulation design involving 50 traits, up to five causal variant per trait per locus. **e.** Statistical power and FDR of *ColocBoost* and HyPrColoc in simulation designs adopted in HyPrColoc with 10 traits and four different causal configurations of 10 traits (codenamed “3+3+2+2”, “5+5”, “5+5+1rand”, “5+5+1rand+1rand” with definitions provided in **Supplementary Note S.6**). **f.** Statistical power and FDR of *ColocBoost* and HyPrColoc in a fully colocalized scenario where multiple causal variants affects all traits, involving 5,10, and 20 phenotypes with up to five true causal variants. **g.** Comprehensive comparison of type I error rate of *ColocBoost* and HyPrColoc under a null scenario with no causal variants across any trait. The error bars in panels **b**-**g** represent 95% confidence intervals. Numerical results for these analyses are reported in **Supplementary Data**.

**Figure S3. Additional metrics to benchmark performance of *ColocBoost* with other multi-trait colocalization methods. a**. Statistical power and FDR of all methods based on the single top-ranked variant, involving 2, 5,10, and 20 phenotypes with up to five true causal variants, with genotype data and induced colocalization configurations designed to mimic real xQTL datasets (**Methods**). **b.** Statistical power and FDR comparison of *ColocBoost* and OPERA for GWAS targeted colocalization, evaluated at the gene level, under the same simulation design used in OPERA. **c.** Statistical power and FDR comparison and **d.** variant-level precision-recall curves by varying the colocalization score threshold in a simulation design incorporating trait-trait correlations across 2, 5,10, and 20 phenotypes with up to five true causal variants. **e.** Statistical power and FDR of *ColocBoost* and HyPrColoc assuming diagonal LD matrix and imposing a single-causal-variant assumption without LD-proximity smoothing in *ColocBoost*. **f.** Statistical power and FDR for single trait fine-mapping analysis (*FineBoost*) with up to five causal variants using SuSiE and *FineBoost* (the single-trait version of *ColocBoost*) in simulation scenarios comprising up to five causal variants. Error bars in panels **a**-**f** represent 95% confidence intervals. Numerical results are reported in Supplementary Data.

**Figure S4. Hyperparameter sensitivity and robustness analysis for *ColocBoost*.** Performances of *ColocBoost* with different hyperparameters are evaluated under the simulation designs involving 2, 5, and 10 traits, up to five causal variants per trait per locus, with genotype data and induced colocalization configurations designed to mimic real xQTL datasets. **a**. Simulation studies to benchmark different choice of probabilistic simplex $\delta^{k}$ used in proximity smoothing in *ColocBoost*. **b**. Sensitivity analysis of $\lambda\in[0,1]$ that controls the strength of proximity smoothing in *ColocBoost*. **c.** Sensitivity analysis of threshold to determine the equivalence across best updates. (**d, e**). Sensitivity of two parameters related to D-SEC, code-named *coloc_thresh* and *func_compare*. **f**. Evaluation of performance for different weight fudge factor $\nu$. (**g, h**). Robustness against weak spurious signals removing criterions. **i**. Robustness of correlation between confidence sets. See detailed definitions of each parameters in **Supplementary Note S.5.2**. Numerical results are reported in **Supplementary Data**.

**Figure S5. Extended *ColocBoost* xQTL analysis in aging human brain.** *ColocBoost* was applied to 17 gene-level cis-xQTL datasets from the aging brain cortex of ROSMAP subjects. **a**. UpSet plot summarizing the top 80 colocalized events in xQTL-only *ColocBoost* analysis across 17 molecular traits. **b.** UpSet plot displaying colocalization patterns across 3 molecular modalities (expression, splicing, and protein abundance) restricted to 3,655 genes with data available for all modalities. **c.** The fraction of cell-type specific fine-mapping (measuring as 95% credible sets) for different brain cell types, that are recovered by cell-type specific colocalizations separately. **d.** Summary of cell-type specific and cell-type shared colocalization events from (left penal) xQTL colocalization analysis spanning 17 molecular traits, and (right panel) eQTL colocalization analysis spanning eQTL data from 6 brain cell types and 4 bulk tissues. The diagonal entries indicate the number of cell-type specific colocalizations and off-diagonal entries indicate the number of colocalizations shared between each pairs of cell types. **e.** Excess-of-overlap (EOO) analysis of the cell-type specific colocalization of eGenes with cell-type gene programs from external brain single-cell RNA-seq data. Numerical results are reported in **Supplementary Data**.

**Figure S6. Functional enrichment of colocalized variants** **a.** Excess-of-overlap (EOO) analysis of “mappable” CoS (number of colocalized variants $\leq3$) and other 95% CoS with cV2F score and RegulomeDB annotations. **b.** Enrichment analysis of variants comprising “mappable” CoS are significantly enriched in regulatory functional annotations from baseline-LD v2.2, including enhancer, promoter, histone modifications, genomic features, conservation, and other annotations. Error bars denote 95% confidence intervals. Numerical results are reported in **Supplementary Data**.

**Figure S7. Properties of genes with multiple causal variants. a.** Scatter plot of the change in log-likelihood between primary CoS and a secondary CoS, where each point is a primary-secondary CoS pair for the gene. **b.** A boxplot summarizing the relationship between the number of colocalized traits and log likelihood changes. **c.** Functional annotation enrichment comparison between high MaxVCP scored variants (MaxVCP>0.5) in primary CoS versus secondary CoS, including annotations from enhancer, promoter, coding, and repressed categories. **d**. An example case of xQTL-only colocalization in gene *ARSB* showing two CoS, one colocalized among all brain cell types and the other colocalized across microglia, astrocytes and oligodendrocytes only. Error bars denote 95% confidence intervals. Numerical results are reported in **Supplementary Data**.

**Figure S8. xQTL-only *ColocBoost* for eQTLs across 13 brain tissues in GTEx.** **a.** UpSet plot summarizing the top 50 colocalized events in xQTL-only *ColocBoost* analysis across 13 brain tissues in GTEx. **b.** CoS-level overlap across ROSMAP xQTL colocalization events, ROSMAP eQTL colocalization events, and GTEx colocalization events involving brain cortex (3 tissues). **c.** Properties of genes with multiple causal variants, including log likelihood changes between primary and secondary CoS, the relationship between the number of colocalized traits and log likelihood changes, and the number of pairs of primary and secondary CoS across brain tissue eQTLs. Error bars denote 95% confidence intervals. Numerical results are reported in **Supplementary Data**.

**Figure S9.** **Validation of *ColocBoost* colocalization.** **a.** Variant set-level excess-of-overlap (EOO) analysis of (i) 95% CoS-gene links from xQTL-only *ColocBoost*, (ii) standard marginal xQTL-gene associations, merged across all 17 xQTL datasets and (iii) 95% credible set (CS)-gene links from SuSiE fine-mapping, merged across all 17 xQTL datasets, against 118,389 regulatory element-gene maps from Promoter Capture Hi-C assay in the dorsolateral prefrontal cortex brain tissue, which profiles targeted 3D contacts linking gene promoters to proximal or distal regulatory elements. (**b, c**) EOO analysis of 95% CoS-gene links across (b) 3 different brain cortical regions and CD14+/CD16- bulk monocytes, and (c) 6 different brain cell types, against regulatory element-gene maps from PCHi-C data. **e**. Colocalization for *SUOX* in detail, also validated by CRIPRi in K562 cells. Error bars denote 95% confidence intervals. Numerical results are reported in **Supplementary Data**.

**Figure S10. Extended disease heritability analyses of variant-level functional annotations derived from *ColocBoost*.** **a.** Correlation matrix of five functional annotations based on the MaxVCP scores from *ColocBoost*. **b.** Heritability enrichment and standardized effect sizes, meta-analyzed across all, brain-related and blood-related traits, conditional on 97 baseline-LD v2.2 annotations of the 5 versions of xQTL MaxPPH derived from HyPrColoc. **c.** Standardized effect sizes, conditional on the 97 baseline-LD v2.2 annotations + MaxVCP-xQTL and CoS‐xQTL annotations (joint $\tau^{*}$). **d.** Standardized effect sizes, conditional on the 97 baseline-LD v2.2 annotations + MaxVCP-xQTL and binarized MaxVCP-xQTL scores at different binarization thresholds (joint $\tau^{*}$). **e.** Heritability enrichment and standardized effect sizes (marginal $\tau^{*}$ and joint $\tau^{*}$) of MaxVCP-xQTL against MaxPIP annotation from SuSiE fine-mapping analysis. **f.** EOO analysis of MaxVCP-xQTL in the disease fine-mapped variants (PIP>0.95) from 94 UK Biobank traits and 930 Million Veteran Program (MVP) traits. **g.** Heritability enrichment and standardized effect sizes (marginal $\tau^{*}$ and joint $\tau^{*}$) of MaxVCP-xQTL annotations for cell‐type‐specific variants and cell‐type‐shared variants. Error bars denote 95% confidence intervals. Numerical results are reported in **Supplementary Data**. Asterisk denotes FDR<5% is computed by Bonferroni correction of the p-values for the number of annotations tested in the S-LDSC analysis.

**Figure S11.** **Extended evaluations of GWAS-xQTL colocalization from *ColocBoost* and competing methods.** **a.** Precision-recall analysis comparing CoS-gene links from AD-xQTL *ColocBoost*, COLOC-Union, pairwise-*ColocBoost*-union, and a version of AD-xQTL *ColocBoost* limited to AD fine-mapped variants (*ColocBoost*-finemapped-GWAS) against enhancer-gene links predicted by ENCODE-rE2G across 354 biosamples. Error bars along both axis indicate 95% confidence intervals. **b**. Precision-Recall for the comparison with enhancer-gene links for each eQTL. COLOC-union only identified colocalization events for a few xQTL, and is excluded from the figure due to a lack of reliable standard error estimation. Manhattan plot of the MaxVCP functional annotation scores of variants from **c.** Pairwise-*ColocBoost*-union and **d.** COLOC-union, with labeled genes containing variants with MaxVCP>0.5. Microglia contributions are highlighted in green. Error bars denote 95% confidence intervals. Numerical results are reported in **Supplementary Data**.

**Figure S12.** **Additional summary of genome-wide GWAS-xQTL colocalization results.** **a.** UpSet plot of colocalization events and identified by AD-xQTL *ColocBoost*. **b.** Manhattan plot of MaxVCP scores, with labeled genes containing variants with MaxVCP>0.1, and microglia contributions are highlighted in green. **c.** Excess overlap of genes showing colocalization in microglia with three microglia specific gene-sets as benchmarks. Error bars denote 95% confidence intervals. Numerical results are reported in **Supplementary Data**.

**Figure S13.** **Example cases of xQTL colocalizations in known AD risk genes.** **a.** Example of established AD gene *BIN1* across multiple molecular contexts and **b**. example of colocalized variants spanning multiple genes *CR1, CR2, YOD1*. One variant, rs669515 (MaxVCP=0.755), colocalized with *CR2* in DLPFC bulk eQTLs, *YOD1* in oligodendrocytes, and *CR1* across oligodendrocytes and all three additional bulk cortical eQTLs. Numerical results are reported in **Supplementary Data**.

**Figure S14.** **Additional details of colocalizations in gene *CTSH***. **a**. Marginal association p-values (at -log10 scale) and **b.** estimated multi-trait coefficients by *ColocBoost*, including all molecular events colocalized. Numerical results are reported in **Supplementary Data**.
